# Integrating multi-session transcranial direct current stimulation with routine physical therapy to improve quadriceps strength and activation in athletes during subacute recovery following ACL reconstruction: A double-blind RCT

**DOI:** 10.1371/journal.pone.0345947

**Published:** 2026-06-11

**Authors:** Naeemeh Haddadi Esfahani, Mohammad Mohsen Roostayi, Volga Hovsepian, Alireza Akbarzadeh Baghban, Zahra Sadat Rezaeian, Shapour Jaberzadeh

**Affiliations:** 1 Department of Physiotherapy, School of Rehabilitation, Shahid Beheshti University of Medical Sciences, Tehran, Iran; 2 Physiotherapy Research Center, Department of Physiotherapy, School of Rehabilitation, Shahid Beheshti University of Medical Sciences, Tehran, Iran; 3 Department of Exercise Physiology, Faculty of Sport Sciences, University of Isfahan, Isfahan, Iran; 4 Proteomics Research Center, Department of Biostatistics, School of Allied Medical Sciences, Shahid Beheshti University of Medical Sciences, Tehran, Iran; 5 Musculoskeletal Research Center, Rehabilitation Research Institute and Department of Physical Therapy, Faculty of Rehabilitation Sciences, Isfahan University of Medical Sciences, Isfahan, Iran; 6 Monash Neuromodulation Research Unit (MNRU), Department of Physiotherapy, Monash University, Melbourne, Australia; Beijing Sport University, CHINA

## Abstract

**Objective:**

Anterior cruciate ligament reconstruction (ACLR) is often associated with persistent quadriceps weakness and impaired voluntary activation. Integrating active anodal transcranial direct current stimulation (a-tDCS) with routine physical therapy (RPT) may offer additional neuromodulatory benefits during the subacute phase of rehabilitation in nonprofessional male athletes.

**Methods:**

Twenty male nonprofessional athletes (mean age: 27.50 ± 7.24 years, BMI: 25.83 ± 3.49) were randomly assigned to two groups: active M1 a-tDCS + RPT (n = 10) and sham M1 a-tDCS + RPT (n = 10). Both groups received 10 sessions of a-tDCS during RPT. The active group received 2 mA stimulation for 20 minutes per session, while the sham group received 2 mA for 30 seconds, followed by ramp-down and discontinuation. Quadriceps strength was measured using an isokinetic dynamometer, voluntary activation was assessed using the superimposed burst technique (Central Activation Ration (CAR) method) Baseline measurements were taken after 10 weeks of RPT, prior to the combined intervention phase.

**Results:**

The active a-tDCS group demonstrated a significant improvement in CAR compared to the sham group (mean difference: 6.85%; 95% CI: 1.92–11.77; p = 0.01), while the sham group showed a slight, non-significant decrease (2.54%; p = 0/80). Within-group analysis revealed significant voluntary activation improvements in the active group (p = 0.005), with no significant changes in the sham group (p = 0.59). No significant between-group differences in quadriceps strength were observed (p = 0.4), although both groups showed significant within-group improvements (active: 21.8%, P ≤ 0.001; sham: 14.8%, p = 0.02), likely due to the effects of RPT.

**Conclusion:**

The integration of multi-session a-tDCS with RPT during the subacute phase of ACLR recovery significantly enhanced voluntary activation and knee-specific function, as measured by the International Knee Documentation Committee (IKDC) score. These findings suggested that multiple-channel a-tDCS may facilitate neural activation and functional recovery, with clinically meaningful improvements in IKDC scores observed in the active group.

Trial Registration: Iranian Registry of Clinical Trials (IRCT) Registration Number: IRCT20231113060048N1; Registered on December 28, 2023. URL: https://irct.behdasht.gov.ir/search/result?query=IRCT20231113060048N1

## Introduction

Anterior cruciate ligament (ACL) injuries are among the most common musculoskeletal injuries in athletes, often requiring surgical reconstruction to restore knee stability and functional mobility [1,2]. In the United States, approximately 100,000 ACL reconstruction (ACLR) surgeries are performed annually, with the associated costs exceeding $2 billion [3]. Despite surgical and rehabilitative advancements, many patients experience persistent quadriceps weakness, which remains a significant barrier to full recovery and return to athletic performance [2–4]. Quadriceps weakness is also associated with increased risk of reinjury and the development of post-traumatic osteoarthritis [2,5,6].

A major contributor to quadriceps weakness post-ACLR is neural inhibition [2,7], involving alterations in spinal reflexes and corticospinal pathways [8–10]. These changes in neural excitability may persist beyond the early postoperative period and potentially contribute to long-term neuromuscular impairments and an increased risk of reinjury [2,10]. Research has shown that corticospinal pathway alterations can emerge as early as two weeks post-surgery [8,11] and continue despite routine rehabilitation efforts [8,10].

Following ACLR, reduction in corticospinal excitability and quadriceps strength are frequently reported, contributing to compromised knee stability and delayed functional recovery [2]. Given the critical role of the quadriceps in joint stabilization, and the association between muscle weakness and poor rehabilitation outcomes [2,5,12,13], early interventions, aimed at restoring quadriceps function, are essential.

While routine rehabilitation protocols often focus on spinal reflex stimulation [8], interventions targeting corticospinal pathways, which may directly address the underlying neural deficits, have received less attention [8–10]. Recent research on transcranial direct current stimulation (tDCS) has highlighted its potential to enhance corticospinal excitability and facilitate motor recovery post-injury [14,15].

One of the mechanisms believed to underpin this effect is Hebbian learning, where simultaneous activation of neurons strengthens their connections, enhancing synaptic efficiency [16]. Additionally, long-term potentiation plays a crucial role in synaptic plasticity, increasing the strength of neural connections with repeated stimulation [17]. These processes are thought to contribute to post-injury cortical reorganization, which is vital for recovery, particularly following ACL reconstruction (ACLR).

Evidence suggests that this therapeutic method can improve muscle strength in both healthy athletes and individuals with neurological conditions, including stroke and multiple sclerosis [14,18,19]. tDCS has been recognized as a safe and effective neuro-modulatory technique, typically utilizing low-intensity currents (1–3 mA) to enhance corticospinal excitability [17,18].

To date, limited research has investigated the application of a-tDCS during the subacute phase following ACLR, particularly in relation to corticospinal deficits and quadriceps inhibition. While Murphy et al. [20] recently demonstrated modulation of intracortical excitability in ACLR patients, their study initiated stimulation as early as two weeks post-surgery. In contrast, our protocol begins at 10^th^ week post-operation; the timing has been selected to minimize confounding impact of pain and fear of re-injury during maximal voluntary contractions, and to ensure graft safety during isometric strength testing. This study aimed to address that gap by introducing a neuromodulatory intervention at the earliest clinically permissible time point for isometric quadriceps strength assessment, ensuring graft integrity and patient safety. Although tDCS has been previously explored in ACLR populations [20], its clinical impact during the subacute phase remains uncertain. Therefore, the objective of this study was to evaluate the effects of anodal tDCS (a-tDCS) on corticospinal excitability, quadriceps activation, and strength following ACL reconstruction. We hypothesized that a-tDCS, when combined with routine physical therapy (RPT), significantly enhances corticospinal excitability and improves quadriceps activation and strength compared to sham stimulation, without causing any adverse effects or safety concerns.

The original protocol specified quadriceps strength assessment at week 8 post-ACLR. However, the evaluation was conducted at week 10 to enhance measurement reliability and minimize the influence of early postoperative factors. Previous studies have shown that early strength testing (weeks 4–8) may yield inaccurate results due to pain, swelling, and arthrogenic muscle inhibition, which compromise voluntary muscle activation and measurement validity [5,21]. Week 10 post-ACLR was selected as the earliest safe time point for quadriceps strength assessment, ensuring that patients could perform maximal voluntary contractions without pain, fear, or risk to graft integrity.

Furthermore, the Central Activation Ratio (CAR), a widely accepted and validated measure of voluntary quadriceps activation, was included in this study to enable a more comprehensive and objective assessment of central motor drive during maximal isometric contraction following ACLR [3,22].

By modulating underlying neural impairments, a-tDCS may enhance rehabilitation outcomes and reduce long-term healthcare burdens, including the need for revision surgeries. Early modulation of corticospinal tract excitability during the subacute phase post-ACLR has the potential to improve quadriceps strength and activation, accelerate return-to-sport timelines, reduce the risk of reinjury, and alleviate persistent functional limitations. Murphy et al. [23] emphasized moving beyond traditional exercise paradigms, and recognizing the complexity of neuromuscular impairments in pain and injury populations. Building on this foundation, we proposed neuromodulatory strategies such as tDCS as adjuncts to rehabilitation. This approach was intended to inform cost-effective, evidence-based protocols, improving outcomes and resource utilization.

## Materials and methods

### Patient and public involvement

Patients and the public were not involved in the design, conduct, or reporting of this trial.

### Trial design

**Fig 1:**
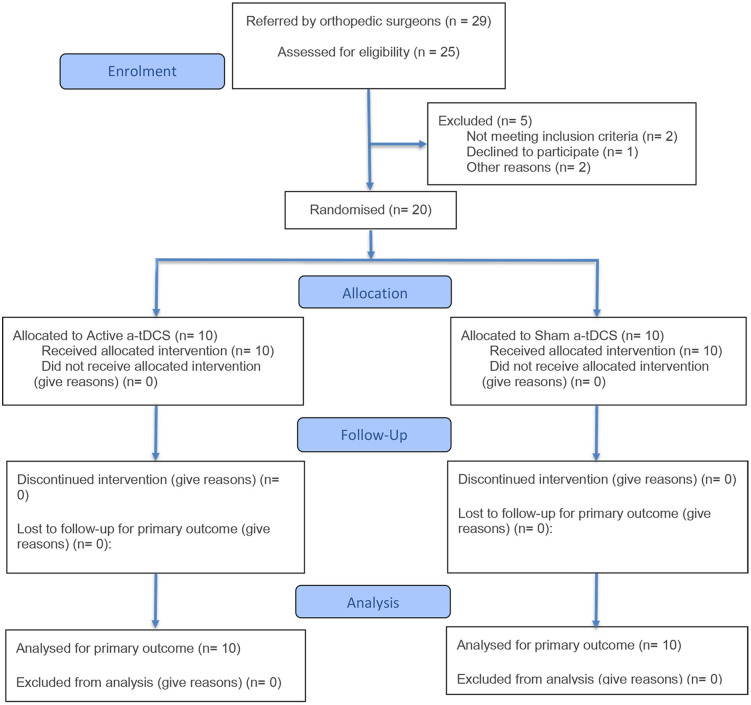
Flow diagram of screening, exclusion, and randomization in the ACLR.

This double-blind randomized clinical trial was designed as a parallel-group study with a 1:1 allocation ratio, using a superiority framework to compare the effects of multiple sessions of active and sham a-tDCS on quadriceps strength and activation in athletes following ACLR. Participants were recruited consecutively following ACLR surgery using convenience sampling. The tDCS intervention was initiated after the 10-week rehabilitation period and baseline assessments, and was delivered over 10 sessions concurrently with RPT. The authors confirm that all ongoing and related trials for this intervention are registered. The participant flow through the trial is presented in Fig 1.

### Trial setting

The study was conducted at a single outpatient rehabilitation center (Hasti Physical Therapy Center) located in Isfahan, Iran, between December 30, 2023 and February 16, 2025. After completing 10 weeks of RPT, participants underwent baseline assessments immediately before the first session of active or sham a-tDCS. All participants in both groups received routine rehabilitation care, including RPT sessions, as part of their post-ACLR recovery. No additional treatments or medications were administered during the trial period.

### Eligibility criteria

No specific eligibility criteria were applied to the site beyond standard clinical infrastructure required for physical therapy and neuromodulation procedures. Participant recruitment was based on the study’s inclusion criteria, targeting individuals who had undergone ACLR and were otherwise healthy, with no history of previous knee surgery, lower extremity injury within the last six months (excluding the ACLR), or any systemic diseases affecting neuromuscular function (e.g., cardiovascular conditions, stroke, or neurological disorders). Participants with intracranial metal clips, cochlear implants, balance disorders, or active skin infections were excluded [22]. Individuals who had undergone meniscectomy or partial medial meniscus tear at the time of ACLR were included, provided they exhibited no clinical signs or symptoms of meniscal injury during testing [24]. ACL reconstruction surgeries were performed by two experienced orthopedic surgeons, following a standardized surgical technique.

Although the original protocol specified inclusion of hamstring autograft recipients only, logistical constraints and graft availability led to the inclusion of both allograft and autograft cases. This change was necessary to ensure recruiting adequate number of participants due to limited access to hamstring grafts at the study site.

Participants included non-professional male athletes involved in volleyball, basketball, or football who had undergone ACLR. These athletes were aged between 18 and 40 years. Participants were classified as non-professional athletes according to the American College of Sports Medicine guidelines [25], engaging in three or more sessions a week structured physical activity for at least 60 minutes per session. They had consistently maintained this level of activity for a minimum of three consecutive years, without receiving financial compensation or holding formal professional affiliation.

### Inclusivity in global research

Additional information regarding the ethical, cultural, and scientific considerations specific to inclusivity in global research is included in the Supporting Information (S3 File).

### Ethical approval and trial registration

The study was approved by the Institutional Review Board. The protocol was registered in the Iranian Registry of Clinical Trials (IRCT), as part of the WHO trial registry network. Written informed consent was obtained from all participants after they received detailed information about the study’s purpose, procedures, and potential risks and benefits. They were given 72 hours to decide about participation and were assured that refusal would not affect their medical care.

### Ethical considerations

Two orthopedic surgeons assisted in referring potential participants who had undergone ACLR. Throughout the study period no harms or unintended events, including those related to tDCS application, physical therapy sessions, or laboratory assessments, were reported in either group.

### Interventions and comparator

#### Rehabilitation Protocol (RPT).

Rehabilitation intervention began approximately 10 weeks after ACLR. During this period, all participants completed a RPT program that was consisted of three sessions per week during the first three weeks, followed by two sessions per week for the remaining nine weeks. Each session lasted approximately 70–90 minutes.

#### Weeks 0–4: early-phase physiotherapy.

The focus during this phase was on graft protection, reducing inflammation, restoring full knee extension, and providing patient education. Pain management included ice therapy [21,26], while functional electrical stimulation and transcutaneous electrical nerve stimulation facilitated voluntary quadriceps activation [8,27]. Exercises during this phase included patellar mobilization, heel strikes, quadriceps sets**,** pelvic tilts, hamstring and gastrocnemius stretches, and straight leg raises. Weight-bearing was introduced gradually, with progression based on individual tolerance [21].

#### Weeks 4–6: progression phase.

During weeks 4–6, the focus shifted to restoring a normal gait pattern, maintaining knee extension, progressing knee flexion, and ensuring graft integrity [21]. Exercises included stationary biking [21], toe raises, balance training, hamstring curls, 4-way hip strengthening, aquatic gait training, and wall slides progressing to mini-squats [28]. Core stability and pool-based exercises were also incorporated [21].

#### Weeks 6–10: strengthening and endurance phase.

During these weeks, the focus was on achieving full range of motion, improving strength and endurance, enhancing proprioception, and avoiding graft overloading [28]. Exercises included stretching, stair climbing, proprioceptive tasks, knee extensions, single-leg squats, and leg presses [21,28]. Throughout the intervention, participants attended the PT center twice weekly. At the conclusion of the final session, follow-up clinical examination by the surgeon confirmed the absence of infection or other complications.

#### tDCS protocol.

**Fig 2:**
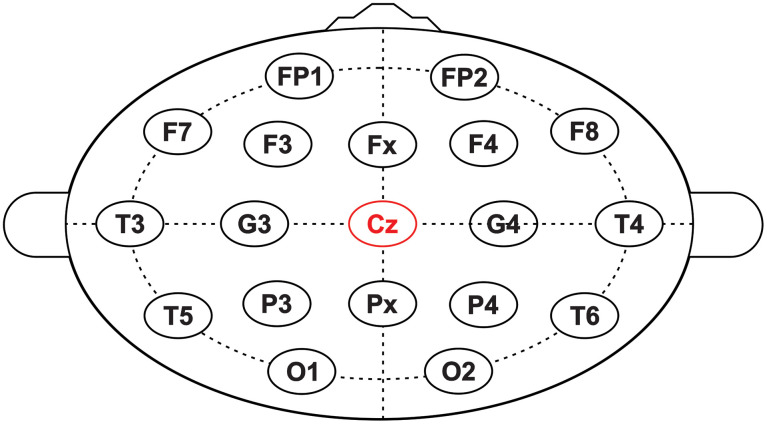
Scalp landmarks and Cz electrode placement for tDCS over the lower limb motor cortex.

Following baseline assessments, participants underwent 10 sessions of tDCS using a battery-operated TES-2CH device (Medinateb), administered three times per week during the first two weeks and twice per week during the final two weeks. Stimulation targeting the lower limb motor cortex was delivered via electrode placement at Cz—corresponding to the M1 leg area—according to the international 10–20 EEG system [14,29–31]. Cz is anatomically defined as the central scalp point—at the vertex—where two reference lines intersect: one from the nasion (between the eyebrows) to the inion (the bony prominence at the lower rear of the skull), and the other connecting the left and right preauricular points. While some studies used C3 or C4 [10,17], Cz offers more direct access to the midline motor representation of the lower limbs [29,31] (Fig 2).

**Fig 3:**
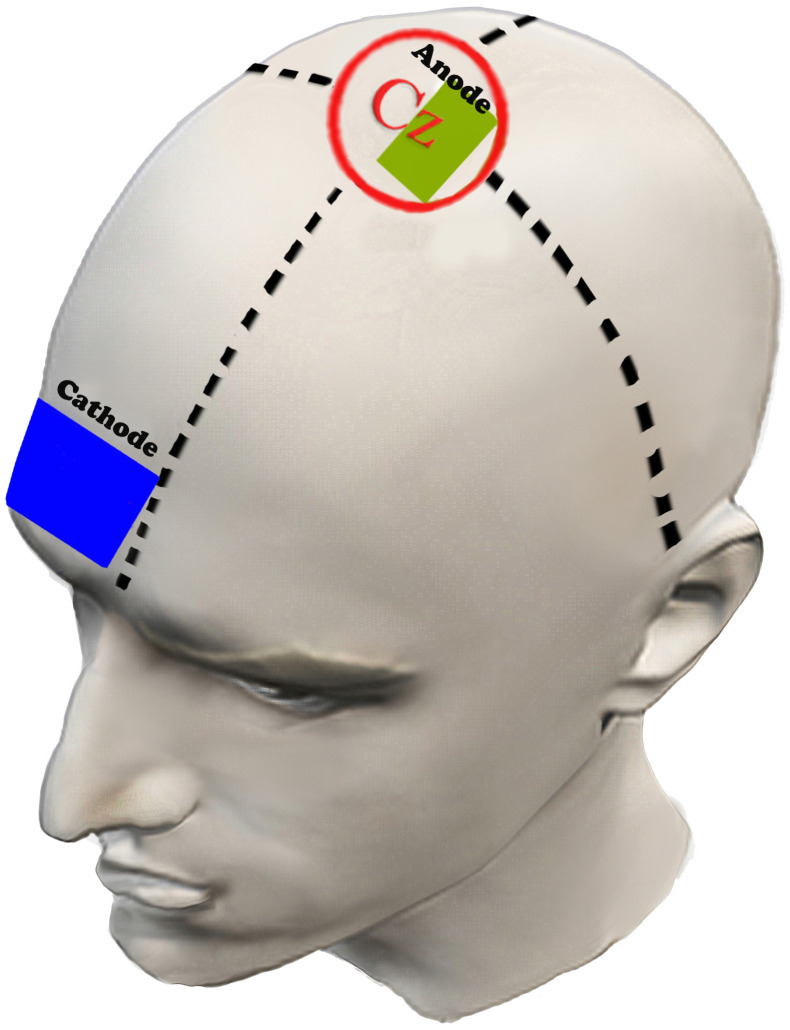
tDCS electrode montage: anode over Cz, cathode over ipsilateral supraorbital region.

Accordingly, in the current study, the anodal electrode (24 cm² carbon) was positioned over Cz (M1 leg area) [14], and the cathodal electrode (35 cm² carbon) was placed over the contralateral supraorbital region [15]. A constant current of 2 mA was delivered during each session and was well-tolerated by all participants (Fig 3).

To reduce skin impedance, electrode sites were moistened and pads were soaked in physiological saline before being affixed with Velcro straps [17]. The active a-tDCS aimed to modulate excitability of the primary motor cortex (M1) and enhance corticospinal output [14,15,18]. Sham stimulation mimicked the active setup by using identical electrode placement, stimulation parameters, and session duration. Although the current was ramped down to zero after 30 seconds in the sham group, electrode contact and device settings were maintained for the full 20-minute session [15]. Participants were blinded to group allocation throughout the intervention, and sensory experiences (e.g., tingling, itching) were monitored to ensure similarity between groups.

To ensure consistency across conditions, both active and sham protocols were delivered using identical electrode placements and stimulation parameters, including current intensity and duration. The stimulation was administered via a battery-driven, constant-current stimulator with a maximum output of 2 mA [15,17]. Participants were seated comfortably in a quiet room and instructed to remain relaxed and minimize movement during stimulation.

The intervention group received active a-tDCS targeting the primary motor cortex (M1) alongside 10 sessions of RPT, while the control group received sham a-tDCS and the same RPT protocol.

After completing 10 weeks of RPT, participants underwent baseline assessments immediately before the first session of active or sham a-tDCS. These included a personal information questionnaire, the International Knee Documentation Committee (IKDC) [32,33], ACL-Return to Sport after Injury (ACL-RSI) [34], Knee injury and Osteoarthritis Outcome Score (KOOS) [35,36], and the Visual Analogue Scale (VAS) as well as laboratory measurements of quadriceps strength and voluntary activation (assessed via the central activation ratio: CAR). All the assessments were repeated after completion of the tenth a-tDCS session.

All the assessments were conducted at the Nilforoshan Laboratory, Faculty of Physical Education, University of Isfahan, before and after the intervention. Devices were calibrated prior to the study and rechecked at each session per manufacturer guidelines. Evaluations were performed at baseline (10 weeks post-ACLR) and after 10 sessions of active or sham a-tDCS combined with RPT. Each combined session of a-tDCS and RPT lasted approximately 80–100 minutes.

No formal eligibility criteria (e.g., certification level or years of experience) were applied to individuals delivering the interventions, but all personnel were trained in the study protocol and proficient in their respective roles.

All participants in both groups received routine rehabilitation care, including RPT sessions, as part of their post-ACLR recovery. No additional treatments or medications were administered during the trial period.

### Outcomes

Baseline assessments were conducted prior to the active a-tDCS or sham interventions, and follow-up measurements were obtained after the completion of the intervention sessions. The baseline evaluation was scheduled at 10^th^ week post-surgery although we committed to recruit volunteers the 8^th^ weeks post-surgery. This timing, week 10 instead of the originally planned week 8, was selected to enhance measurement reliability and reduce the confounding effects of early postoperative factors such as pain, swelling, and arthrogenic muscle inhibition.

#### Patient-reported outcome measures.

At both time points (baseline and post-intervention assessments), participants completed patient-reported outcome measures evaluating knee pain, function, overall activity, and readiness to return to sport. These included a demographic form, VAS, and the Persian versions of the KOOS, IKDC and ACL-RSI. The KOOS questionnaire evaluated symptoms (KOOS Sx), quality of life (KOOS QoL), pain (KOOS Pain), and activities of daily living (KOOS ADL). Given the participants’ limited engagement in sports activities during the recovery phase, the KOOS Sport subscale was excluded from statistical analysis. To provide a comprehensive understanding of recovery, both physical and psychological assessments were integrated into the study protocol.

In addition to physical recovery and functional stability, psychological and social elements play a crucial role in recovery and return-to-play outcomes after ACLR [37]. It has been established that inadequate mental preparation is a significant risk factor for graft failure following ACLR [38]. Key psychological factors influencing an athlete’s ability to return to play include psychological readiness, motivation, fear of re-injury, and self-confidence [39]. Consequently, this study employed the Persian version of the ACL-Return to Sport after Injury (ACL-RSI) questionnaire [34] to evaluate athletes’ psychological readiness, emphasizing aspects such as emotional state, performance confidence, and risk perception concerning their return to sports activities.

#### Laboratory outcomes.

**Fig 4:**
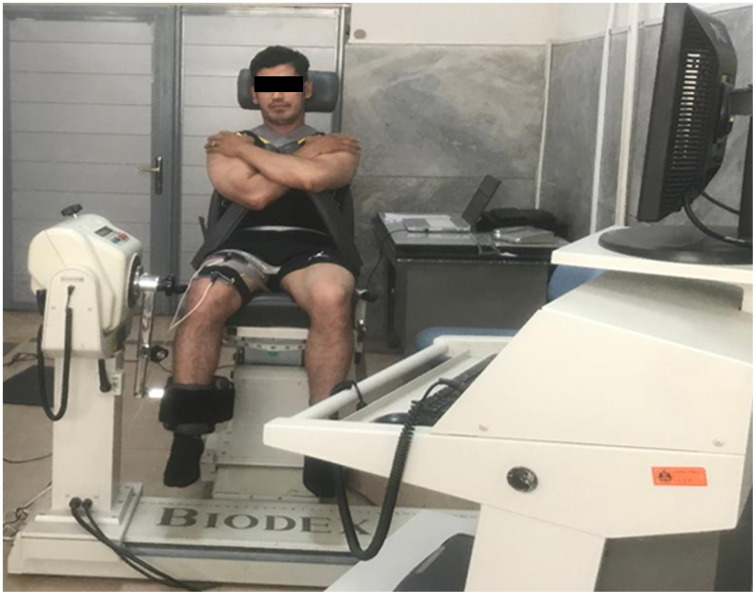
Participant positioning during MVIC assessment using isokinetic dynamometry.

Isometric quadriceps strength was evaluated by measuring the Normalized Peak Torque (NPT) generated during MVIC, using an isokinetic dynamometer (Biodex System 3; Biodex Medical Systems, Inc., Shirley, NY). Participants completed a 5-minute warm-up on a cycle ergometer, followed by standardized positioning on the dynamometer with both hip and knee flexed at 90°. Stabilization straps were applied across the waist and shoulders to prevent trunk movement, and the tibia was secured to the dynamometer arm using Velcro just above the ankle [22]. Participants kept their arms crossed over the chest throughout testing (Fig 4).

To familiarize participants with the testing protocol, they performed two submaximal isometric quadriceps contractions (25%, 50%, and 75% of perceived effort), each lasting 2–4 seconds [40]. Submaximal electrical stimulation was simultaneously applied using two 20 cm² carbon electrodes placed on the proximal and distal quadriceps. A 10-pulse train (600 μs duration, 100 Hz, 125 V) was delivered via a Grass S48 stimulator with an SIU8T isolation unit [22]. These trials were for familiarization only and not included in the analysis.

In the subsequent phase of the study, participants performed three to four MVICs of the quadriceps during knee extension. Each contraction was sustained for 6 seconds, with standardized verbal encouragement provided to optimize effort [22]. A rest period of at least 90 seconds was allowed between trials to minimize fatigue and ensure measurement accuracy [41]. Once torque output plateaued, supramaximal electrical stimulation was applied using the superimposed burst (SIB) technique to recruit additional motor units not voluntarily activated by the participant [40]. No feedback was given during SIB trials. The highest torque value recorded across the seven trials (with and without stimulation) was used for analysis. All torque values were normalized to body mass and expressed as Newton meters per kilogram (Nm/kg).

A commonly used method to assess quadriceps activation failure following ACLR is the calculation of the CAR [22]. CAR is computed by dividing the maximal voluntary torque immediately before the superimposed electrical stimulation by the peak torque generated during the stimulation [42]. This ratio reflects the degree of central activation and quantifies the extent of voluntary neural activation. The mathematical expression of CAR is presented in equation (1) [3].


$$\begin{array}{c} 𝐜𝐚𝐥𝐜𝐮𝐥𝐚𝐭𝐢𝐨𝐧\;𝐨𝐟\;𝐂𝐀𝐑:\;𝐂𝐀𝐑=\;[𝐌𝐕𝐈𝐂\;𝐭𝐨𝐫𝐪𝐮𝐞/\;(𝐌𝐕𝐈𝐂+𝐒𝐈𝐁\;𝐭𝐨𝐫𝐪𝐮𝐞)]\;\times 100 \end{array}$$ (1)

### Harms

All participants tolerated the 2-mA stimulation without discomfort or adverse sensations, and no participant reported unusual perceptions during or after the sessions. Sensory experiences were systematically assessed using a post-session questionnaire, in which participants rated the intensity of sensations such as tingling and itching on a 0–10 scale. These sensations remained within a mild range, and no cases of burning or intolerance were reported.

#### Sample size and power calculation.

At the time of study design, no published studies had investigated the combined effects of a-tDCS and RPT on voluntary activation and quadriceps strength in ACLR patients. Murphy et al. (2024a) [20], which was published after our study was designed, examined quadriceps strength but did not assess voluntary activation. Therefore, sample size estimation was based on a pilot study involving 10 participants. Power analysis was conducted using conventional parameters (α = 0.05, β = 0.80), with estimated effect sizes of 1.63 for voluntary activation and 1.1 for normalized peak torque during maximum voluntary isometric contraction (MVIC). A total of 20 male athletes were recruited accordingly. Although the observed variability in MVIC data was higher than anticipated, pilot-based sample size was considered adequate for detecting meaningful changes in both voluntary activation and MVIC. Nevertheless, the statistical power for MVIC may have been slightly reduced due to increased data dispersion. No interim analyses were performed, and no formal stopping guidelines were established.

### Randomisation

#### Sequence generation.

The randomisation sequence was generated using simple randomisation without any restrictions such as blocking or stratification, by an independent individual not involved in recruitment, assessment, or treatment.

#### Allocation concealment mechanism.

Based on this sequence, group labels were pre-assigned and placed into sequentially numbered, opaque, sealed envelopes prepared in advance.

#### Implementation.

This procedure, involving an independent allocator unaware of the sequence and uninvolved in other study aspects, ensured full concealment of group assignment until intervention initiation.

#### Blinding.

To minimize the risk of outcome assessment bias, both participants and outcome assessors were blinded to group allocation and intervention type. Routine physiotherapy and preparation for a-tDCS sessions were offered by a trained physiotherapist who was also one of the study investigators. Device settings for active or sham stimulation were configured by an independent, trained technician who was not involved in the study, based on the group allocation concealed in sealed envelopes. Laboratory measurements (MVIC and voluntary activation) were performed by a trained researcher blinded to group assignment. The same trained physiotherapist, who remained blinded to group allocation throughout the intervention, assessed pain (VAS), knee function (IKDC, KOOS), and psychological readiness (ACL-RSI). Blinding was maintained throughout, with all personnel involved in intervention delivery and outcome assessment unaware of group assignment.

### Statistical analyses

All statistical analyses were conducted using SPSS software (version 18; IBM Corp., Armonk, NY, USA). The Shapiro–Wilk test was applied to assess the normality of data distribution prior to selecting appropriate statistical tests.

Between-group comparisons (active a-tDCS vs. sham a-tDCS) were performed using independent t-tests for normally distributed variables and the Mann–Whitney U test for non-normally distributed data. Within-group comparisons (pre- vs. post-intervention) were analyzed using paired-sample t-tests or Wilcoxon signed-rank tests, depending on the distribution of the data. Effect sizes were calculated using Cohen’s d, along with 95% confidence intervals (CIs), to determine the magnitude of observed changes. Power analysis was conducted using G*Power 3.1.9.7 freeware (Released March 17, 2020, University of Düsseldorf, Düsseldorf, Germany) [43].

No missing data were observed during the study; therefore, no imputation was performed, and all analyses were conducted using complete cases. All 20 participants who completed the intervention protocol were analyzed according to their assigned group (active or sham a-tDCS), consistent with a per-protocol approach. The pre-specified primary outcomes were voluntary activation and quadriceps strength, measured as normalized peak torque during MVIC. Secondary outcomes included functional, pain, and psychological scores, specifically KOOS, IKDC, VAS, and ACL-RSI. No additional analyses, including subgroup or sensitivity analyses, were prespecified or conducted post hoc. All statistical evaluations were limited to the predefined primary and secondary outcomes.

## Results

Among 29 male athletes referred to the study, 25 were assessed for eligibility between December 30, 2023 and February 16, 2025. Five participants were excluded prior to randomization: two due to prior knee surgeries (ACLR), one due to perceived concerns about procedural risks after reviewing the written study details, one because of a recent traffic accident involving the knee joint, and one who relocated to another city prior to data collection. None of these five individuals received any part of the intervention. Consequently, 20 athletes were randomized. All of them received the allocated intervention as planned and completed the study without dropout. All participants were included in the final analysis, with no post-randomization losses or exclusions (adherence rate = 100%). In practice, active or sham a-tDCS was delivered by a trained technician and routine physiotherapy by a licensed physiotherapist, as planned. All participants adhered fully to the intervention schedule, and no deviations from the protocol were observed. All participants in both groups received RPT throughout the trial as part of standard care, with no additional concomitant treatments. No adverse events or unintended effects were reported in either group.

### Demographic and baseline characteristics

All 20 randomized participants completed the baseline assessment and were included in the analysis. Of these, 14 had undergone ACLR using allografts, and 6 received hamstring autografts. There were no significant differences between the active a-tDCS + RPT (n = 10) and sham a-tDCS + RPT (n = 10) groups in terms of demographic characteristics or baseline outcome measures (P > 0.05). Data were available for all participants at all outcome time points, and no missing data were reported. Detailed baseline characteristics are presented in Table 1.

**Table 1:** Demographic characteristics and baseline data for the active and sham groups.

Variable	(Mean±SD)			P (between- group)
	Active a-tDCS + RPT	Sham a-tDCS + RPT	Total	
**Age (years)**	28.00 ± 7.20	27.00 ± 7.70	27.50 ± 7.20	0.57
**BMI (kg/m2)**	25.67 ± 3.27	25.99 ± 3.87	25.83 ± 3.49	0.88
**Height (cm)**	181.60 ± 6.10	177.40 ± 5.00	179.50 ± 5.80	0.11
**Weight (kg)**	84.60 ± 10.90	81.90 ± 13.60	83.30 ± 12.00	0.43
**Voluntary Activation (CAR, %)**	94.62 ± 3.70	94.90 ± 4.30	94.76 ± 3.90	0.76
**Normalized Peak Torque (NPT) (Nm/kg)**	2.43 ± 0.79	2.03 ± 0.66	2.23 ± 0.74	0.23
**Graft type (Allograft/Autograft)**	7/3	7/3	14/6	–

**Abbreviations: CAR=Central Activation Ratio, used to assess voluntary activation; NPT=Normalized Peak Torque.**

### Voluntary activation

**Fig 5:**
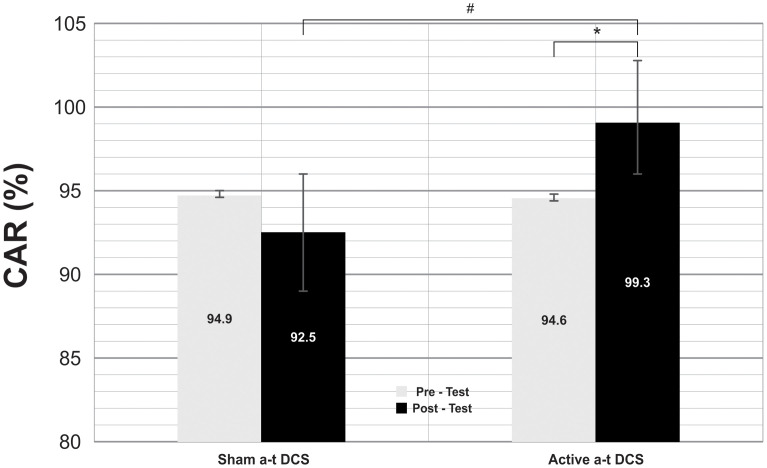
Mean voluntary activation (assessed via CAR) pre- and post-intervention in active and sham groups. * P < 0.05 indicates a significant within-group difference; # P < 0.05 indicates a significant between-group change. Abbreviations: tDCS: Transcranial Direct Current Stimulation.

Post-intervention, the active a-tDCS group showed a significant voluntary activation increase (+4.98%, p = 0.01), compared to the sham group, which exhibited a slight decrease of 2.54% with no significant change (p = 0.76). Within-group analysis confirmed a significant improvement in the active group (mean change: 4.7; 95% CI: 1.94–7.49; p = 0.005; Cohen’s d = 1.8), whereas the sham group showed no meaningful change (mean change: −2.4; 95% CI: −7.15–2.33; p = 0.59). Voluntary activation changes are illustrated in (Fig 5). Note: Although the term “voluntary activation” is used in the text, Fig 5 illustrates CAR-based values, which represent the standard metric used in similar studies.

### Quadriceps Strength (as NPT during MVIC)

**Fig 6:**
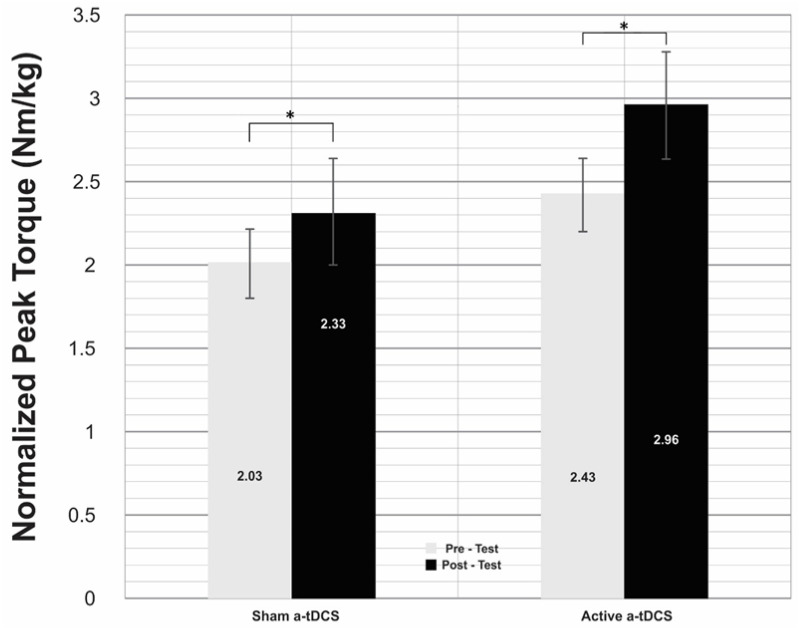
Mean normalized peak torque (%) before and after intervention in active and sham a-tDCS groups. *P<0.05 indicates a significant within-group change. Abbreviations: tDCS: Transcranial Direct Current Stimulation.

No baseline difference in MVIC was found between groups (P = 0.25). Post-intervention, both showed significant within-group improvements. The active a-tDCS group demonstrated a greater increase (mean change: 0.65 Nm/kg; 95% CI: 0.50–0.81; P < 0.01; Cohen’s d = 0.64, + 21.81%), while the sham group also improved (mean change: 0.40 Nm/kg; 95% CI: 0.15–0.65; P = 0.02; Cohen’s d = 0.41, + 14.79%). Although the primary analysis for this group was non-parametric, the confidence interval was reported here based on the corresponding t-test output for descriptive purposes only. Although the between‑group difference was 0.63 Nm/kg, the Standard Error of Measurement (SEM) was 11.9 Nm/kg in the active group and 7.9 Nm/kg in the sham group. This comparison shows that the observed difference is much smaller than the typical measurement error of the instrument. As a result, part of this difference may reflect normal variability inherent to the test. However, this does not diminish the relevance of the findings; rather, it highlights the need to interpret small between‑group differences with consideration of the instrument’s sensitivity. The between-group and within-group changes in NPT during MVIC are illustrated in (Fig 6).

#### Patient-reported outcomes: pain, function, and psychological readiness.

At baseline, no significant differences were found between the active and sham groups across all measured variables (p > 0.05). Post-intervention, outcomes remained similar between groups, except for IKDC, which showed a significant difference (p = 0.03).

Within-group analysis revealed significant improvements in both groups. The active group showed significant improvements in KOOS Sx and QoL (p < 0.001), while the sham group also demonstrated statistically significant gains (P = 0.007 and P = 0.025 respectively). Both groups exhibited meaningful changes in KOOS pain, ADL, IKDC, VAS, and ACL-RSI (all p < 0.05). Full data are shown in Table 2.

**Table 2:** Patient-reported outcomes (pain and disability) in the active and sham groups.

Parameter	Measurement Phase	Active a-tDCS + PT	Sham a-tDCS + PT	Mean of Total Sample	Difference (95% CI)	Cohen’s d	P (between group)
**KOOS Sx**	**Pre**	66.90 ± 20.64)	64.80 ± 15.28	65.85 ± 17.71	−14.96-19.16	0.12	0.80
	**Post**	78.30 1 ± 6.15	79.00 ± 16.48	78.65 ± 15.88	−16.03-14.63	−0.06	0.93
	**Within Group Mean Difference (95% CI)**	7.29- 15.51	5.07- 23.33				
**KOOS Qol**	**Pre**	33.80 ± 9.37	33.60- (15.99)	33.70 ± 12.76	−12.11-12.51	0.02	0.97
	**Post**	42.50 ± 8.80	41.10 -(17.05)	41.80 ± 13.23	−11.35- 14.15	0.1	0.82
	**Within Group Mean Difference (95% CI)**	6.36- 11.04	1.17- 13.83				
**KOOS Pain**	**Pre**	72.90 ± 13.72	73.10 ± 17.07	73.00 ± 15.08	−14.75-14.35	−0.01	0.85
	**Post**	82.50 ± 9.66	79.70 ± 17.45	81.10 ± 13.80	−10.45-16.05	0.2	0.97
	**Within Group Mean Difference (95% CI)**	2.27-10.93	5.92-13.28				
**KOOS ADL**	**Pre**	77.60 ± 17.32	77.30 ± 18.95	77.45 ± 17.67	−16.76-17.36	−0.02	0.80
	**Post**	90.50 ± 7.68	83.20 ± 17.86	86.85 ± 13.89	−6.07-20.67	0.53	0.63
	**Within Group Mean Difference (95% CI)**	3.95-21.85	3.68-8.12				
**IKDC**	**Pre**	40.50 ± 5.84	42.80 ± 4.66	41.65 ± 5.27	−7.26-2.66	0.44	0.34
	**Post**	53.60 ± 3.44	50.10 ± 3.35	51.85 ± 3.76	0.31-6.69	1.03	0.03
	**Within Group Mean Difference (95% CI)**	11.06-15.14	4.87-9.73				
**VAS**	**Pre**	2.30 ± 0.67	1.70 ± 0.67	2.00 ± 0.73	−0.034-1.23	0.90	0.09
	**Post**	1.00 ± 0.67	0.80 ± 0.92	0.80 ± 0.92	−0.55-0.95	0.25	0.44
	**Within Group Mean Difference (95% CI)**	−1.65-(−0.95)	−1.13-(−0.67)				
**ACL_RSI**	**Pre**	57.00 ± 10.77	53.25 ± 9.93	55.13 ± 10.26	−5.98-13.48	0.36	0.39
	**Post**	70.08 ± 12.16	59.42 ± 11.05	64.75 ± 12.56	−0.25-21.58	0.92	0.06
	**Within Group Mean Difference (95% CI)**	7.71-18.45	0.59-11.75				

**Abbreviations: KOOS = Knee injury and Osteoarthritis Outcome Score; Sx = Symptoms; QoL = Quality of Life; ADL = Activities of Daily Living; IKDC = International Knee Documentation Committee; VAS = Visual Analogue Scale; ACL-RSI = ACL–Return to Sport after Injury.**

#### Correction for multiple comparisons and sensitivity analyses.

To account for the risk of type I error due to multiple testing, Bonferroni correction was considered for the two pre-specified primary outcomes (Voluntary Activation and NPT), resulting in an adjusted significance threshold of P < 0.025. Under this criterion, voluntary activation remained statistically significant in both the between-group (P = 0.01) and within-group (P = 0.005) comparisons. For NPT, the between-group comparison was not significant even before correction (P = 0.40), but within-group improvements remained statistically significant in both the active (P = 0.000) and sham (P = 0.022) intervention groups, meeting the adjusted threshold. Secondary outcomes, including KOOS, VAS, ACL-RSI, and IKDC, were interpreted in an exploratory manner. Among these, only the between-group comparison for IKDC yielded a p-value (0.030) that did not meet the adjusted threshold, and was therefore interpreted with caution.

Due to the small sample size and potential limitations of normality testing, non-parametric tests were additionally performed to confirm the robustness of statistical interpretations. Applying these tests did not alter the interpretation of statistical significance in any of the outcomes.

Change scores for voluntary activation (Δvoluntary activation = post–pre) were calculated for all participants. Normality was assessed using the Shapiro–Wilk test and was violated in both groups (P < 0.05). Therefore, a non-parametric Mann–Whitney U test was conducted to compare Δvoluntary activation between the intervention and sham groups. The results indicated a significant between-group difference (U = 9.00, P = 0.001), which remained significant after Bonferroni correction (adjusted α = 0.025), reinforcing the reliability of the effect.

Change scores for IKDC (ΔIKDC = post–pre) were calculated for all participants. The Shapiro–Wilk test indicated a non-normal distribution in the intervention group (P < 0.05), while the sham group followed normal distribution. Given this violation, a non-parametric Mann–Whitney U test was applied to compare ΔIKDC between groups. The analysis revealed a statistically significant difference (U = 13.00, P = 0.004).

## Discussion

This study investigated whether multiple sessions of active a-tDCS, applied alongside RPT, could enhance recovery during the subacute phase following ACLR. The active a-tDCS group demonstrated significant improvements in voluntary activation (P = 0.01), indicating enhanced motor cortical excitability. However, no significant between-group differences were observed in other outcomes, such as MVIC and patient-reported outcomes, including knee function (KOOS), psychological readiness (ACL-RSI), and pain levels (VAS), except for the IKDC scores (p = 0.03).

These findings suggest that although active a-tDCS may modulate neural mechanisms underlying muscle activation, its short-term impact on broader clinical outcomes remains limited. Among the assessed measures, only IKDC scores showed statistically significant improvement, suggesting potential benefits in knee-specific functional recovery. In contrast, other outcomes, such as MVIC, KOOS, ACL-RSI and VAS, did not demonstrate significant between-group differences, indicating that the current a-tDCS protocol may not yet elicit clinically meaningful improvements as perceived by patients. Nevertheless, the observed enhancement in voluntary activation supports the hypothesis that a-tDCS can positively influence central neural mechanisms and may serve as a complementary tool in post-ACLR rehabilitation protocols.

Interestingly, voluntary activation deficits are also reported in other musculoskeletal conditions such as knee and hip osteoarthritis [44,45]. While our findings are specific to ACLR rehabilitation, the observed neuro-modulatory effects of a-tDCS may have broader relevance for populations experiencing similar central activation impairments. Further research is warranted to explore such applications. For example, preliminary studies in individuals with knee osteoarthritis have also demonstrated beneficial effects of tDCS on both pain and functional outcomes [46,47], further supporting its broader potential in musculoskeletal rehabilitation.

To facilitate interpretation, the discussion is now clearly divided into five sections: (1) demographic and baseline characteristics, (2) laboratory-based outcomes: MVIC, (3) laboratory-based outcome: voluntary activation, (4) psychological readiness (ACL-RSI), and (5) self-reported functional and psychological outcomes.

### 1. Demographic and Baseline Characteristics

The demographic and baseline characteristics did not differ significantly between the two groups, indicating successful randomization and minimizing the risk of selection bias. This lack of statistically significant differences enhances the interpretability of post-intervention findings by reducing the likelihood that observed effects were due to pre-existing group disparities.

### 2. Laboratory-Based Outcome: MVIC

No statistically significant between-group differences were observed in MVIC of quadriceps strength. Although quadriceps strengthening remains a cornerstone of ACLR rehabilitation [2,8], persistent muscle weakness cannot be solely attributed to post-surgical atrophy [48]. However, the meta-analysis by Birchmeier and colleagues [48] underscores the need for targeted interventions, as weakness often persists despite conventional protocols. Neurophysiological evidence further implicates central neural inhibition as a key contributor to post-ACLR deficits [8,12,49], with reduced corticospinal excitability contributing to impaired MVIC and muscle activation [8,12]. These neural alterations may endure for years following surgery, limiting functional recovery [8,12].

Based on these findings, we had anticipated the improvement of MVIC as a secondary outcome of stimulating the M1 leg area, especially given the significant increase observed in the voluntary activation. This expectation was grounded in the theoretical potential of a-tDCS to enhance motor cortical excitability and consequently facilitate greater neural drive to the quadriceps muscle. The primary motor cortex (M1) contains pyramidal neurons that influence the activation of alpha motor neurons, which are directly responsible for voluntary muscle contraction [49]. Therefore, enhanced excitability of these neurons through tDCS is thought to augment motor output and potentially lead to increased muscle activation and quadriceps strength.

Despite the theoretical rationale for a-tDCS, several factors may account for the absence of significant between-group differences in MVIC. Muscle strength is governed not only by neural activation [15] but also by structural and mechanical properties, including muscle cross-sectional area [13,17], fiber type distribution, and tendon stiffness. Although the observed increase in corticospinal excitability likely enhanced motor unit recruitment and synaptic transmission [17], these neural adaptations may have been insufficient to overcome peripheral limitations such as reduced muscle mass, suboptimal fiber composition, and tendon compliance—factors that typically require sustained mechanical loading and time to remodel [13,50].

Importantly, the intervention was conducted during the subacute rehabilitation phase, a period in which high-load resistance training is generally contraindicated to avoid surgical complications. As a result, conditions necessary for muscle hypertrophy and architectural remodeling were not present, limiting the potential for strength gains despite central neural facilitation.

Additionally, while MVIC is a valid measure of voluntary strength [22], it may not fully capture functional improvements. Dynamic strength, coordination, or task-specific neuromuscular adaptations might be better assessed through performance-based tests (e.g., hop tests), which were not included due to post-surgical restrictions. This is consistent with previous critiques of isokinetic testing, which highlight its limited functional relevance and lack of transferability to sport-specific movements [51]. Therefore, while neural enhancements were observed, their functional implications remain to be fully elucidated.

Finally, neuroplastic mechanisms such as long-term potentiation and Hebbian learning—where repeated, coordinated neuronal activity strengthens synaptic connections—are believed to drive cortical reorganization and motor relearning [17], both essential for restoring strength and function after ACLR. To translate these adaptations into meaningful functional gains, additional task-specific training and repetitive stimuli may be required, which may have been insufficient in our protocol. Future research combining a-tDCS with resistance training or neuromuscular re-education could better leverage these neuroplastic processes for more effective rehabilitation.

It is also important to consider that, although the between‑group difference in quadriceps strength was not statistically significant, the observed mean difference remained far below the SEM in both groups. This indicates that the magnitude of the difference falls within the normal measurement variability expected for isometric torque assessments. Consequently, the absence of a statistically significant effect should be interpreted with an understanding of the inherent variability and limited sensitivity of MVIC measurements, rather than as evidence of a lack of meaningful change. It is well established that isometric torque outcomes are influenced by multiple sources of fluctuation, including participant effort, neuromuscular activation, and mechanical factors, all of which contribute to variability in repeated assessments. Furthermore, quadriceps strength is shaped by a combination of neural, structural, and biomechanical determinants, and these interacting influences may be particularly pronounced during the subacute rehabilitation phase. Overall, these considerations suggest that the small between‑group difference observed in our study likely reflects the interplay of these factors and the measurement characteristics of the assessment tool.

### 3. Laboratory-Based Outcome: Voluntary Activation

Voluntary activation was quantified using the CAR, a widely used method that estimates the extent of motor unit recruitment during MVIC. Previous studies have reported a range of voluntary activation values (measured via CAR) in the ACLR limb [3,8,50,52]. In healthy individuals, these values are generally reported to exceed 95% [53–56]. In fact, older active individuals aged 66–83 demonstrated significantly lower voluntary activation levels (95.5% activation) compared to younger individuals (98.1% activation) [54].

The present findings align with existing research, suggesting that diminished strength and impaired voluntary activation of the quadriceps are frequently observed following ACLR. Reduced voluntary activation may persist for up to six months after surgery despite RPT interventions [8], and in some cases, it may continue even years postoperatively [2].

CAR values in our participants averaged 94.76 ± 3.9 prior to the intervention, consistent with previously reported CAR ranges (88.8 ± 9.1 to 94.6 ± 2.7 and 91.2 ± 6.2) observed approximately 6–7 months after ACLR [8,50,52]. Following the intervention, the active group demonstrated a notable increase in CAR (99.34), whereas the sham group exhibited no significant improvement, with CAR decreasing from 94.90 to 92.49. The statistically significant difference in voluntary activation levels between the two groups (P ≤ 0.05) supports the hypothesis that active a-tDCS can enhance motor cortical excitability. This finding also met the Bonferroni-adjusted significance threshold (P < 0.025), confirming the robustness of the result. Importantly, the between-group difference was not only statistically significant but also demonstrated a large effect size (Cohen’s d = 1.8), indicating a substantial magnitude of change. In contrast, the sham group showed a decline in CAR, underscoring the importance of targeted interventions to prevent further deterioration.

### 4. Psychological Readiness (ACL-RSI)

It has been observed that psychological responses prior to surgery and during early recovery have been associated with the likelihood of returning to the pre-injury level of sport within 12 months [1,57]. Therefore, along with physical rehabilitation, psychological recovery following ACL injury and reconstruction may be crucial. The ACL-RSI questionnaire serves as a valid tool for assessing a patient’s psychological readiness to return to sports after ACLR [1,34]. This underscores the need for a dual-focus approach in rehabilitation, addressing both physical and psychological domains to optimize return-to-sport outcomes.

In this study, both active and sham a-tDCS groups showed significant within-group improvements in ACL-RSI scores and quadriceps strength following the intervention. However, the absence of significant between-group differences suggests that these gains were primarily attributable to RPT, with no added benefit from a-tDCS in enhancing psychological readiness or strength outcomes.

According to previous studies, increased strength in the quadriceps is associated with improved psychological well-being and a greater preparedness to resume physical activities [58]. This interdependence between neuromuscular recovery and psychological resilience is well-documented. No significant increase in quadriceps strength was observed following active a-tDCS treatment compared to sham a-tDCS, and similarly, ACL-RSI scores did not show significant improvement in this study. This outcome aligns with previous research that indicates a positive correlation between quadriceps strength and psychological readiness. Given the positive correlation between quadriceps strength and psychological readiness, the lack of increase in quadriceps strength likely contributed to the unchanged psychological readiness in this study, as assessed by the ACL-RSI questionnaire.

A large-scale study involving 681 athletes reported progressive improvements in ACL-RSI scores over time, with values rising from 41.3 ± 25.4 preoperatively to 55.1 ± 21.3 at four months post-ACLR [1]. Our findings are consistent with this trajectory: the sham group demonstrated a score of 59.42 ± 11.05 at four months post-surgery, while the pre-intervention score was 55.12 ± 10.26. Notably, the active a-tDCS group showed a higher post-intervention score of 70.08 ± 12.16.

The lack of a statistically significant difference may also be explained by several factors. These include the insufficient duration or intensity of a-tDCS sessions, which may have limited their potential to produce measurable effects. Future studies should consider optimizing stimulation parameters, including current intensity, electrode montage, and session frequency, to better evaluate the potential of a-tDCS in psychological recovery. Additionally, the strong placebo effect observed in the sham group could have masked any additional benefits of active a-tDCS.

This discrepancy may also stem from the multifaceted nature of psychological recovery. Returning to sport involves more than physical strength; it requires overcoming fear of reinjury, rebuilding confidence, and enhancing mental resilience [39]. These psychological skills are best developed through real-field rehearsals, which introduce competition-induced stresses and challenge an individual’s balance and coordination. Such exercises are typically avoided in the early stages of post-surgery rehabilitation (first three months). This approach is consistent with the Aspetar clinical practice guideline, which emphasizes that early rehabilitation should prioritize pain management, restoration of range of motion, and gradual strength recovery, while delaying sport-specific and psychologically demanding tasks until later phases [21].

Moreover, restoring confidence in bearing weight on the injured limb is a gradual process. Exercises targeting overall and knee-specific proprioception and coordination in real-field settings are crucial for overcoming the fear of reinjury. These exercises were excluded from the current study’s protocol to minimize the risk of reinjury during the early rehabilitation phase. By the third month post-surgery, weight-bearing is typically permitted; however, real-field exercises that simulate sports-specific movements were avoided to ensure a safe recovery and reduce the risk of re-injury [21].

### 5. Self-Reported Functional and Psychological Outcomes

We hypothesized that patient-reported outcomes, including pain, function, and psychological readiness, would improve following the tDCS intervention. However, after the intervention, the active and sham groups demonstrated similar outcomes, with no significant differences in KOOS, VAS, and ACL-RSI scores, except for IKDC, which showed a statistically significant change. Both groups demonstrated statistically significant within-group improvements from pre- to post-intervention across several outcomes, irrespective of the stimulation condition.

The fact that only the IKDC score showed a statistically significant improvement, while the KOOS did not, may be explained by the distinct focus and sensitivity of these two outcome measures. The IKDC score primarily assesses functional performance and the ability to engage in knee-specific tasks such as walking, running, and pivoting, which are more directly influenced by physical rehabilitation interventions like RPT and active a-tDCS. Consequently, improvements in these physical capabilities may lead to significant changes in IKDC scores within a relatively short timeframe. In contrast, the KOOS includes a broader range of factors, including pain, quality of life, and activities of daily living, as well as psychological aspects such as anxiety and concerns about reinjury. Because of this wider scope, KOOS may be less sensitive to the subacute stage physical improvements observed in this study. Moreover, changes in psychological recovery, which are typically slower to manifest, may not have been adequately captured in the timeframe of the study, which may have contributed to the absence of significant improvement in KOOS scores.

As for the VAS scores, the lack of a significant difference may be explained by the low initial pain levels reported by participants. With a baseline pain intensity of only 3 (on a 10-point scale), there was limited potential for significant pain reduction following the intervention. Additionally, while tDCS is known to influence motor cortical excitability and muscle activation, its analgesic effects may be more evident in populations with moderate to severe pain, and therefore may have been less pronounced in this study. The placebo effect within the sham group may have obscured any potential improvements in pain levels, as both groups may have experienced some level of pain relief regardless of the active intervention.

### Limitations

While our sample size was calculated based on pilot data to detect between-group differences in MVIC, the variability observed in this outcome was greater than expected. As a result, we may have been underpowered to detect statistically significant differences.

Furthermore, MVIC captures only static strength and may not adequately reflect dynamic or sport-specific improvements, which are more relevant to athletic performance. Given the subacute stage of recovery during the intervention period, and the inherent limitations of functional assessment at this phase, inclusion of dynamic performance measures was neither feasible nor clinically appropriate. Although validated functional tests such as hop tests or single-leg squats are typically introduced from the fourth month onward, their relevance to the subacute phase remains limited. Future research may focus on developing and integrating adapted functional assessments that are both safe and meaningful for early rehabilitation, without compromising the emphasis on neuro-modulatory strategies. While the surgical technique was standardized across participants, the study included only male participants and used hamstring tendon autografts and allografts; therefore, the findings may not generalize to female athletes or to individuals receiving other graft types or undergoing different surgical approaches. Caution is advised when generalizing these results to other populations, such as older adults or those undergoing different rehabilitation protocols.

## Conclusion

This study demonstrated that multi-session of a-tDCS, when combined with RPT during the acute postoperative phase, significantly reduced motor cortex inhibition and enhanced corticospinal excitability. These neurophysiological effects were reflected in improved CAR and IKDC scores, suggesting a beneficial role for a-tDCS in accelerating knee-specific functional recovery following ACLR. Future research should involve larger, well-powered trials incorporating a broader range of functional performance measures.

## Supporting information

S1 File S1 Protocol. (DOCX)

S2 File Completed Inclusivity Questionnaire. (DOCX)

S3 File Revised-04.CONSORT 2025-Checklist. (DOCX)
