# Supplementary material for: Integrating multi-session transcranial direct current stimulation with routine physical therapy to improve quadriceps strength and activation in athletes during subacute recovery following ACL reconstruction: A double-blind RCT
Source: PLoS One. 2026 Jun 11;21(6):e0345947. doi: 10.1371/journal.pone.0345947 (PMC13257960; doi:10.1371/journal.pone.0345947)
Supplement: S1 File — (DOCX) [file pone.0345947.s001.docx]

| فرم شماره 1ـ پروژه دانشجویی |
| --- |


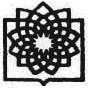


دانشگاه علوم پزشکی شهید بهشتی

دانشکده علوم توانبخشی

### 1ـ اطلاعات مربوط به دانشجو

| نام و نام خانوادگی: نعیمه حدادی اصفهانی | شماره دانشجوئی: |
| --- | --- |
| مقطع تحصیلی: دکتری | رشته: فیزیوتراپی |
| آدرس: اصفهان، خیابان شیخ صدوق شمالی | شماره تلفن: 09131171904 |

### 2ـ عنوان پروژه

| عنوان فارسی: تاثیر افزاینده ی تحریک فراجمجمه ای جریان مستقیم کورتکس حرکتی به فیزیوتراپی روتین بر قدرت عضله چهارسررانی در ورزشکاران با جراحی بازسازی رباط صلیبی قدامی: کارآزمایی بالینی تصادفی دوسو کور  کلمات کلیدی: تحریک فراجمجمه ای جریان مستقیم، جراحی بازسازی رباط صلیبی قدامی، قدرت، عضله چهارسر |
| --- |
| عنوان انگلیسی: The increasing effect of transcranial direct current stimulation of the motor cortex to routine physical therapy on quadriceps muscle strength in athletes with anterior cruciate ligament reconstruction surgery: a double-blind randomized clinical trial.  کلمات کلیدی: Transcranial direct current stimulation, anterior cruciate ligament reconstruction, quadriceps, strehgth |

### 3ـ اطلاعات مربوط به اساتید راهنما و مشاور

#### 1ـ3ـ استاد راهنما

نام و نام‌خانوادگي: دکتر محسن روستایی رتبه علمی دانشگاهی: دانشیار

آخرین مدرک تحصیلی: دکتری رشته تحصیلی: فیزیوتراپی

نشاني: محل کار: خیابان تهران نوـ روبروی بیمارستان بوعلی ـ دانشکده توانبخشی شهید بهشتی ـ گروه فیزیوتراپی. تلفن:

پست الكترونيك: [Roosta@sbmu.ac.ir](mailto:Roosta@sbmu.ac.ir)

### 2ـ3 ـ اساتید راهنما و مشاور

| پست الکترونیک | آدرس و شماره تلفن | رتبه علمی دانشگاهی | رشته تحصیلی | آخرین مدرک تحصیلی | نام و نام خانوادگی | اساتید |
| --- | --- | --- | --- | --- | --- | --- |
| Roosta@sbmu.ac.ir | تهران- دانشکده توانبخشی شهید بهشتی | دانشیار | فیزیوتراپی | دکتری | دکتر محسن روستایی | استاد راهنما |
| shapour.jaberzadeh@monash.edu | Monash University, Victoria, 3199, Australia | استادیار | فیزیوتراپی | دکتری | دکتر شاپور جابرزاده | مشاور اول |
| zrezaeian@yahoo.com | اصفهان ـ دانشکده علوم توانبخشی | استادیار | فیزیوتراپی | دکتری | دکتر زهرا سادات رضائیان | مشاور دوم |

**4. Project Summary**

Anterior cruciate ligament (ACL) rupture is among the most common sports-related injuries. Currently, ACL reconstruction (ACLR) is considered the preferred treatment strategy in most cases, with the primary goals of restoring function, enabling return to sport, and preventing further injury.

However, quadriceps weakness following ACLR is highly prevalent and remains a major limiting factor in clinical recovery—even years after completion of standard rehabilitation. This persistent deficit may be associated with reduced cortical excitability along corticospinal pathways.

Despite the importance of these neurophysiological changes, conventional rehabilitation protocols for ACLR have paid limited attention to the role of corticospinal adaptations in muscle strength recovery. As a result, the effectiveness of routine rehabilitation remains limited in addressing long-term neuromuscular deficits.

Transcranial direct current stimulation (tDCS) is a safe and non-invasive neuromodulation technique that delivers a low-intensity direct current (typically 1–3 mA) to modulate cortical excitability. Depending on the polarity and placement of electrodes, tDCS can either enhance or suppress neuronal activity.

Following ACLR, it is hypothesized that applying tDCS over the primary motor cortex (M1) may increase corticospinal excitability, thereby improving voluntary activation and strength of the quadriceps muscle.

In the present study, adult male athletes with a history of unilateral ACLR in the early postoperative phase will be recruited using a non-random convenience sampling method, based on predefined inclusion and exclusion criteria. Participants will then be randomly assigned to one of two groups: routine physiotherapy combined with anodal tDCS (a-tDCS), or routine physiotherapy combined with sham tDCS (s-tDCS).

The active tDCS intervention will involve placement of the anodal electrode over the M1 and the cathodal electrode over the contralateral supraorbital area. A direct current of 2 mA will be applied for 20 minutes per session, across a total of ten sessions. The aim is to enhance quadriceps muscle strength through increased corticospinal excitability.

Quadriceps strength will be assessed using a dynamometer before and after the a-tDCS intervention. Additionally, participants will complete the following validated outcome measures: KOOS (Knee Injury and Osteoarthritis Outcome Score), IKDC (International Knee Documentation Committee), and the Visual Analog Scale (VAS) for pain.

Enhancing quadriceps muscle strength, as a key parameter, may not only help prevent recurrent injuries but also reduce financial burden and alleviate the psychological concerns commonly experienced by patients following ACLR.

**5. Significance of the Project and Rationale for Selection**

**Section 5.1: Introduction**

Anterior cruciate ligament (ACL) injury is one of the most common knee-related injuries among active individuals and athletes ([1](#_ENREF_1)), with ACL rupture being a prevalent orthopedic condition that occurs at a rate of 30 to 78 cases per 100,000 people annually ([2](#_ENREF_2)). The primary goal of ACLR surgery is to restore ligamentous stability; however, in the long term, a significant number of patients experience poor outcomes, including a reinjury rate exceeding 20% ([3](#_ENREF_3)), and a 50% incidence of osteoarthritis within 15 years post-surgey ([4](#_ENREF_4)).

Quadriceps muscle weakness following ACLR is highly prevalent and has been identified as a major limiting factor in clinical functional recovery, often persisting for years after completion of routine rehabilitation ([5](#_ENREF_5)).

Quadriceps dysfunction may be significantly associated with both objective and self-reported outcomes, increased risk of reinjury, and long-term joint health complications following ACLR ([6](#_ENREF_6)). Although ACLR rehabilitation programs largely focus on restoring quadriceps strength, recent studies have shown that even after completion of routine rehabilitation, the affected limb may exhibit reduced quadriceps strength for up to four years post-surgery compared to the control group (injured side) ([7](#_ENREF_7)). This strength deficit has been reported to exceed 20% when compared to the contralateral healthy limb ([8](#_ENREF_8)).

Evidence suggests that quadriceps dysfunction following ACLR may be associated with neuromuscular impairments, including altered excitability of both spinal reflexes and corticospinal pathways that emerge after surgery ([5](#_ENREF_5)). Routine rehabilitation strategies for ACLR patients are primarily based on stimulating spinal reflex pathways—such as neuromuscular electrical stimulation—while the effects of corticospinal modulation (i.e., cortical stimulation) have received comparatively less attention ([5](#_ENREF_5), [9](#_ENREF_9)).

Corticospinal excitability is not only affected during the early stages of injury but may persist for years following ACLR, potentially contributing to long-term deficits in muscle strength and neuromuscular performance in these patients ([5](#_ENREF_5)).

Transcranial direct current stimulation (tDCS) is a safe and effective method for modulating brain activity, in which a low-intensity direct current (1 to 3 mA) is applied to increase or decrease cortical excitability ([10](#_ENREF_10)). According to previous studies, this therapeutic approach has been shown to enhance muscle strength in healthy athletes as well as in patients with stroke and multiple sclerosis ([11-13](#_ENREF_11)).

Research has also demonstrated reduced corticospinal excitability in patients with ACLR ([14](#_ENREF_14)). Therefore, it appears that stimulating and activating corticospinal pathways through tDCS may improve quadriceps muscle activity.

The aim of this study is to investigate the effect of ten sessions of routine rehabilitation combined with a-tDCS on improving quadriceps muscle activity and reducing its dysfunction. The hypothesis of this study is that a-tDCS stimulates the corticospinal pathway and enhances quadriceps muscle activity in athletes with a history of ACLR.

**5.2 Statement of the Problem**

ACL rupture accounts for more than 50% of all knee injuries ([15](#_ENREF_15)) and is also among the most common sports-related injuries ([16](#_ENREF_16)), affecting over 200,000 individuals annually in the United States. The estimated direct and indirect costs associated with ACL injuries exceed $7 billion per year ([15](#_ENREF_15)). Patients and athletes seeking to return to activity typically undergo ACLR to restore mechanical stability of the knee. Currently, ACLR is considered the preferred treatment strategy in most cases, with the ultimate goal of restoring function, enabling return to sport, and preventing further injury ([17](#_ENREF_17), [18](#_ENREF_18)).

However, recent studies have reported suboptimal outcomes following surgery: only 24% of athletes return to their pre-injury level of sport within one year ([19](#_ENREF_19)), less than 60% within two years ([20](#_ENREF_20), [21](#_ENREF_21)), and fewer than 62% within five years ([22](#_ENREF_22)). Therefore, further research is needed to meet athletes’ expectations regarding surgical and rehabilitation outcomes.

The ACL is not merely a mechanical stabilizer; it also serves as a vital afferent information transmitter. Laboratory studies have shown that during ACL loading, feedback from joint mechanoreceptors is transmitted to gamma motor neurons, which in turn activate an appropriate set of motor units required for muscle contraction ([23](#_ENREF_23)).

Injury to the ACL disrupts this mechanoreceptor feedback mechanism, leading to reduced recruitment of quadriceps motor units and subsequent muscle weakness ([24](#_ENREF_24), [25](#_ENREF_25)). This neural inhibition, which results in diminished voluntary activation of the quadriceps muscle, is referred to as arthrogenic muscle inhibition (AMI), and is believed to function as an internal protective mechanism for recently injured tissues.

Since AMI involves central nervous system feedback, it appears that reduced quadriceps activation and persistent weakness following ACLR may be attributed to ongoing AMI—especially considering that voluntary activation deficits have been observed in both the reconstructed and contralateral limb ([26](#_ENREF_26), [27](#_ENREF_27)).

Therefore, one of the contributing factors to prolonged quadriceps weakness after ACLR is neural inhibition, which involves both spinal reflex pathways and descending corticospinal pathways ([28-30](#_ENREF_28)). Alterations in the excitability of these two pathways have been linked to quadriceps weakness following ACLR ([30-33](#_ENREF_30)).

Two systematic reviews encompassing a total of 27 studies have indicated that rehabilitation interventions should focus on normalizing neural excitability following ACLR in order to effectively address quadriceps dysfunction ([14](#_ENREF_14), [34](#_ENREF_34)). To develop more effective rehabilitation strategies that target impaired neural excitability, it is essential to determine when these potential changes occur in spinal reflex pathways and descending corticospinal tracts after ACL injury and reconstruction, so that treatment plans can be appropriately timed and tailored.

Spinal reflex excitability of the quadriceps can be assessed using the Hoffman reflex normalized to maximal muscle responses (H:M ratio), while corticospinal excitability is measured using transcranial magnetic stimulation, including determination of the active motor threshold ([5](#_ENREF_5)).

Previous studies have shown that corticospinal changes related to the quadriceps muscle may persist in patients following ACL injury and in those with chronic anterior knee pain, with an average duration of approximately 22 months post-injury ([32](#_ENREF_32), [35](#_ENREF_35)). However, research specifically focused on ACLR remains limited. According to the study by Leply et al., spinal reflex excitability appears to be impaired up to two weeks after ACLR but not at six months. Notably, this study only assessed changes in these two neural pathways at week 2 and month 6 post-ACLR. They reported a significant reduction in spinal reflex excitability at two weeks, followed by a recovery and increased activity in this pathway at six months ([5](#_ENREF_5)), which aligns with another study showing increased excitability between one and three months post ACLR ([33](#_ENREF_33)).

It therefore appears that spinal reflex excitability decreases during the early phase of injury and increases between two weeks and three months post-surgery. This period typically coincides with the resolution of pain and swelling, during which patients commonly begin routine rehabilitation programs ([36](#_ENREF_36)). Modalities such as transcutaneous electrical nerve stimulation (TENS) or cryotherapy may be beneficial during this phase to reduce pain and inflammation. However, in later stages—when athletes return to activity—corticospinal deficits may still persist, and interventions aimed at enhancing corticospinal excitability may be more appropriate ([5](#_ENREF_5)).

In other words, after approximately four weeks, when spinal reflex pathways have largely recovered through routine rehabilitation, it may be necessary to address the emerging decline in corticospinal excitability, which typically begins around week two post-surgery.

To enhance corticospinal excitability, a-tDCS can be used as a non-invasive brain modulation technique ([37](#_ENREF_37), [38](#_ENREF_38)). This method involves the application of low-amplitude direct currents through electrodes placed on the scalp to modulate the excitability level of the corticospinal pathway ([10](#_ENREF_10)).

tDCS has commonly been used in the treatment of neurological disorders such as stroke and multiple sclerosis ([11](#_ENREF_11), [12](#_ENREF_12)). More recently, it has emerged as a promising approach for managing musculoskeletal conditions, including post-traumatic osteoarthritis, total knee arthroplasty, and ACL rupture ([39](#_ENREF_39), [40](#_ENREF_40)).

tDCS is an electrical device equipped with two electrodes—anode and cathode—that are placed directly on the scalp and can induce changes in neural excitability. The typical current intensity ranges from 1 to 3 mA and alters the polarization of neuronal membranes ([41](#_ENREF_41)). This modulation affects neuronal excitability and influences brain activity ([42](#_ENREF_42)). The facilitatory or inhibitory effects of stimulation on the brain may persist for several hours after the intervention ([43](#_ENREF_43)).

The device is inexpensive, portable, and battery-powered. Its portability is one of the major advantages of tDCS compared to other techniques such as transcranial magnetic stimulation. Moreover, tDCS is painless and can be applied either before or during motor or cognitive activities ([44](#_ENREF_44)).

Alterations in neuroplasticity and excitability are key pathophysiological factors in many neurological disorders. Therefore, modulating cortical activity through non-invasive stimulation may represent a valuable therapeutic approach. The primary effect of tDCS is a shift in resting membrane potential, which—depending on the polarity of the active electrode—can facilitate or inhibit neuronal activity. In general, anodal stimulation increases cortical excitability, while cathodal stimulation decreases it. The cell body and the initial segment of the axon are more affected by these changes than other cellular regions. Neurophysiological studies using transcranial magnetic stimulation have shown that both anodal and cathodal stimulation can alter corticospinal excitability by up to 40% ([10](#_ENREF_10)).

The mechanism of action of tDCS has been investigated in both short-term and long-term contexts. Short-term effects are primarily observed during the stimulation period itself. Low-intensity direct current alters the resting membrane potential of neurons, thereby influencing spontaneous neural excitability.

Anodal stimulation induces depolarization, which increases the rate of neural activity, whereas cathodal stimulation causes hyperpolarization of the membrane and reduces neural activity. Although the weak current used in tDCS does not directly generate action potentials, laboratory studies have shown that simultaneous changes in the membrane potential of the cell body and axonal terminals may underlie the observed alterations in excitability ([38](#_ENREF_38)).

The long-term effects of tDCS are primarily associated with the induction of long-term potentiation and long-term depression through modulation of N-methyl-D-aspartate (NMDA) receptors ([45](#_ENREF_45)). Pharmacological studies have shown that changes in postsynaptic intracellular calcium concentration are responsible for NMDA receptor modulation ([46](#_ENREF_46)).

Multiple lines of evidence confirm that, in addition to glutamatergic receptors such as NMDA, inhibitory interneurons involving GABA also play a role in the long-term effects of tDCS ([47](#_ENREF_47), [48](#_ENREF_48)). MRI-based measurements have demonstrated that anodal tDCS reduces GABA concentration, which in turn facilitates motor memory and motor learning processes ([49](#_ENREF_49)).

Cortical excitability can be modulated by placing either anodal or cathodal electrodes over the M1, resulting in increased or decreased excitability, respectively ([50](#_ENREF_50)). Theoretically, direct electrical stimulation of the motor cortex leads to immediate enhancement of muscle performance ([51](#_ENREF_51)). Therefore, it seems appropriate to focus on increasing corticospinal excitability after two-month post-ACLR, once routine physiotherapy has been completed.

In ACLR patients, persistent quadriceps weakness often impedes full recovery, and this deficit—along with muscle atrophy—can persist for years following surgery ([8](#_ENREF_8), [28](#_ENREF_28)). Numerous studies have shown that routine physiotherapy alone is insufficient to fully restore quadriceps strength ([5](#_ENREF_5), [8](#_ENREF_8), [52](#_ENREF_52), [53](#_ENREF_53)). This muscle weakness, which results from neural inhibition, contributes to a wide range of complications including reduced knee extension, gait abnormalities, atrophy, dynamic instability, chronic knee pain, and early-onset osteoarthritis ([6](#_ENREF_6), [29](#_ENREF_29), [54](#_ENREF_54)).

Despite these findings, many athletes are expected to return to sport within six months after ACLR ([55](#_ENREF_55)). Therefore, therapeutic approaches that enhance physical performance—while adhering to ethical standards—have gained increasing attention among both clinicians and researchers.

The reason most studies investigate quadriceps muscle performance as a key aspect of recovery after ACLR is due to its critical role in knee stability and its association with numerous short-term and patient-centered outcomes ([56-58](#_ENREF_56)), including functional performance ([5](#_ENREF_5), [59](#_ENREF_59), [60](#_ENREF_60)), self-reported function ([60](#_ENREF_60)), return-to-play rates ([23](#_ENREF_23)), and the risk of ACL re-injury ([56](#_ENREF_56)).

Some studies have examined hamstring muscle performance or cross-sectional area following ACLR, particularly in cases where hamstring autografts were used for reconstruction ([61-64](#_ENREF_61)). Other studies have assessed both quadriceps and hamstring function to monitor post-surgical changes, evaluate rehabilitation progress, and guide return-to-sport decisions after ACLR ([61](#_ENREF_61), [65](#_ENREF_65)). A systematic review reported persistent strength deficits in both knee extensors and flexors beyond six months post-ACLR ([66](#_ENREF_66)). Another review emphasized the importance of quadriceps and hamstring strength by providing reference values for athletes with ACLR in rotational sports such as football, basketball, and handball ([66](#_ENREF_66)).

Since muscular strength is directly linked to physical performance, sport-specific technique, injury prevention, and rehabilitation in athletes ([13](#_ENREF_13)), the present study will focus on quadriceps strength following ACLR. Specifically, this study aims to evaluate the effect of a-tDCS applied over the M1, two months after ACLR in athletes, targeting corticospinal excitability and assessing changes in quadriceps strength using a dynamometer. The hypothesis is that this intervention will enhance corticospinal activation and improve quadriceps strength.

In addition to restoring strength and functional stability as key factors, psychological and social components also play a crucial role in recovery and return to sport following ACLR ([18](#_ENREF_18)). A systematic review with meta-analysis identified low psychological readiness as a risk factor for graft rupture after ACLR ([67](#_ENREF_67)). Psychological readiness for return to sport and activity—including motivation, mood disturbances, recovery expectations, fear of reinjury, and self-confidence—is closely associated with an athlete’s potential to resume sport participation ([68](#_ENREF_68)).

The present study aims to evaluate the Persian version of the ACL-Return to Sport after Injury (ACL-RSI) questionnaire ([69](#_ENREF_69)) to assess athletes’ psychological readiness in terms of emotional response, confidence in performance, and risk appraisal related to return to sport before and after a-tDCS intervention.

Graft type appears to influence knee function following ACLR. Over the past two decades, quadriceps tendon autografts (QTAs) have gained popularity due to their advantages in knee stability and muscle strength recovery ([70](#_ENREF_70), [71](#_ENREF_71)). Patellar tendon autografts (PTAs) and hamstring tendon autografts (HTAs) remain the most commonly used grafts ([72](#_ENREF_72), [73](#_ENREF_73)). HTAs are associated with favorable quadriceps recovery ([74](#_ENREF_74)), but often result in hamstring deficits and slower rehabilitation progression. PTAs offer good hamstring recovery and knee stability, but are commonly associated with anterior knee pain and quadriceps weakness ([74](#_ENREF_74), [75](#_ENREF_75)).

Therefore, graft selection for ACLR remains a subject of debate due to the distinct advantages and disadvantages of each option. A meta-analysis comparing QTAs and HTAs reported significantly better isokinetic hamstring strength with QTAs, with results comparable to PTAs at 3, 6, and 12 months postoperatively. Conversely, HTAs showed significantly better isokinetic quadriceps strength at 6 months and similar outcomes to QTAs at 12 months ([76](#_ENREF_76)). These findings suggest that specific muscle strength deficits following ACLR may be related to the graft harvest site. Given that quadriceps and hamstring strength vary depending on graft type at 6 and 12 months post-ACLR, it is essential to account for graft selection in study design.

As most surgeons utilize hamstring autografts for ACLR, the present study will include athletes who have undergone ACL reconstruction using hamstring tendon autografts. Additionally, since ACL injuries are frequently accompanied by meniscal damage, this factor will also be considered in the sample selection process.

**5.3 Novelty and Rationale of the Study**

Return to sport is the primary goal for athletes following ACLR. However, many athletes resume unrestricted physical activity around months 7 or 8 post-surgery—often before persistent deficits in muscular performance have been resolved. This premature return increases the risk of reinjury, psychological distress, and imposes substantial financial burdens on sports organizations. Therefore, addressing quadriceps strength deficits may offer a valuable opportunity for early and effective intervention.

To date, only one study has investigated the effects of a-tDCS following ACLR. That study examined the immediate impact of a single session of a-tDCS on 10 individuals who were six months post-reconstruction. The results indicated no significant immediate effect on quadriceps muscle activity or subjective pain and symptom scores. The authors recommended future research with larger sample sizes and multiple sessions ([51](#_ENREF_51)).

The present study aims, for the first time, to initiate cortical pathway stimulation via ten sessions of a-tDCS during the early postoperative phase. This approach may help modulate corticospinal excitability before deficits become entrenched. If successful, this intervention could mitigate quadriceps weakness and its associated complications—offering meaningful support to this athletic population and potentially reducing the long-term costs of reinjury and revision surgeries.

**6. Literature Review (literature review)**

In the literature review section, a total of 299 articles were initially retrieved based on the following keywords:

"ACL", "Anterior cruciate ligament", "ACL injury", "ACL rehabilitation", "ACL repair", "ACL injury*", "ACL surgery", "Muscle inhibition", "Muscle strength", "Strength*", "Arthrogenic inhibition", "Arthrogenic muscle inhibition", "tDCS", "Transcranial direct current stimulation", "Noninvasive brain stimulation", "Quadriceps strength".*

After removing duplicate and irrelevant articles, 148 studies were selected, and ultimately 28 articles were reviewed in detail. Among these, 9 relevant studies were categorized into five major thematic groups.

tDCS is commonly used in the treatment of neurological disorders such as Parkinson’s disease, stroke, and others. More recently, it has emerged as a promising approach for managing musculoskeletal conditions ([51](#_ENREF_51)). Consequently, the number of studies focusing on musculoskeletal applications of tDCS remains limited. Since the present study aims to investigate the effect of a-tDCS on quadriceps muscle strength, and due to the scarcity of musculoskeletal-specific literature, two studies conducted on neurological patients that examined the impact of a-tDCS on quadriceps strength were selected and included in this section.

Additionally, one study was included that examined the effects of a-tDCS on pain and muscle strength in individuals with patellofemoral pain syndrome, as well as a study investigating quadriceps strength enhancement in healthy athletes and a related systematic review. Finally, studies focusing on ACL rupture and ACLR were also reviewed.

To support the technical specifications required for the present study’s a-tDCS intervention, two additional articles were included that specifically addressed the characteristics and parameters of tDCS stimulation. These studies provide valuable insights for selecting appropriate stimulation protocols in this research.

**1. Studies Investigating Quadriceps Strength in Neurological Conditions (e.g., Stroke and MS) Before and After tDCS**

Tanaka et al. ([12](#_ENREF_12)) conducted a study in 2011 to examine the effect of a single session of a-tDCS on knee extensor strength and grip force in patients with chronic subcortical stroke. Maximum force of knee extension was used as the primary outcome measure, based on prior evidence linking knee extensor strength to lower limb functional activities such as sit-to-stand, walking, and stair climbing in stroke patients.

Eight participants with chronic subcortical stroke performed knee extension and grip strength tests using their hemiparetic limbs before, during, and 30 minutes after receiving either a-tDCS or sham stimulation s-tDCS. Stimulation was applied over the M1, corresponding to the C3 or C4 region of the affected hemisphere. The anodal electrode (35 cm²) was placed over M1, and the cathodal electrode (50 cm²) was positioned on the contralateral forehead. A current intensity of 2 mA was delivered for 15 minutes. In the sham condition, the same setup was used, but current was applied only for the initial 15 seconds.

Knee extension MF and grip force were assessed using a dynamometer, with four repeated measurements averaged for each time point. Results showed a significant increase of 21 Newtons (13.2%, P < 0.01) in knee extension force during a-tDCS compared to baseline and sham stimulation. This effect lasted less than 30 minutes. No significant change was observed in grip strength.

The authors contrasted their findings with those of Hummel et al. ([77](#_ENREF_77)), who reported improvements in grip strength using 1 mA stimulation. Tanaka et al. hypothesized that the higher current intensity (2 mA) in their study may have more effectively targeted the M1 region associated with lower limb control, which is located deeper within the interhemispheric fissure and is less excitable than the upper limb representation. They also noted that due to the low spatial resolution of a-tDCS, stimulation may have influenced not only the quadriceps but also other lower limb muscle groups such as the tibialis anterior and hamstrings.

These findings underscore the importance of stimulation parameters—particularly current intensity and electrode placement—in modulating corticospinal excitability. Based on this evidence, a 2-mA anodal stimulation over M1 appears necessary to effectively enhance corticospinal excitability and subsequently improve quadriceps strength.

Fietsam et al. ([11](#_ENREF_11)) conducted a study in 2021 to investigate changes in lower limb weakness and gait impairments in individuals with multiple sclerosis following a single session of a-tDCS. One of the earliest symptoms of multiple sclerosis is unilateral limb weakness, particularly affecting the lower extremities. Asymmetry in lower limb strength contributes to progressive deterioration in walking ability, which is considered one of the most significant factors affecting quality of life in people with multiple sclerosis.

Glucose is the primary energy source for skeletal muscle activity. A notable aspect of this study was the use of positron emission tomography (PET) imaging with fluorodeoxyglucose, a glucose analog, to objectively assess multi-muscle activity in the lower limbs during physical exertion. Increased glucose uptake and asymmetry between homologous muscles in both legs were associated with higher metabolic cost of walking, elevated energy demand in the affected limb, and consequently, earlier onset of fatigue ([11](#_ENREF_11)).

This fatigue was attributed to the loss of activity in fatigue-resistant muscle fibers, which led to compensatory recruitment of higher-threshold motor units—resulting in earlier fatigue and increased glucose consumption. The authors suggested that increasing motor unit recruitment and the proportion of activated muscle fibers in the affected limb could reduce glucose uptake asymmetry and improve metabolic efficiency, thereby lowering the energy demand in the more impaired limb.

Despite previous efforts, interventions aimed at correcting strength asymmetries and improving gait performance in people with multiple sclerosis had shown limited effectiveness. Based on this, the hypothesis was proposed that a-tDCS might effectively enhance excitability in the motor cortex region corresponding to the weaker lower limb, thereby promoting more symmetrical muscle activation. The samples were in the age range of 25 to 57. Isokinetic strength testing was first conducted to identify the weaker limb. Participants then received either a single session of a-tDCS or s-tDCS. Stimulation was applied using saline-soaked electrodes (5 × 7 cm) placed over the motor cortex (C3 or C4) corresponding to the weaker limb, with the cathode positioned over the contralateral supraorbital region. A current intensity of 3 mA was delivered for 20 minutes. Following stimulation, participants rested for 10 minutes to allow optimal neuromodulatory effects, and then walked on a treadmill for 20 minutes. Two minutes after treadmill activity, fluorodeoxyglucose was injected, and participants underwent PET imaging.

Results showed that a-tDCS altered glucose uptake patterns and reduced asymmetry indices across various muscle groups in each participant. These findings suggest that a-tDCS improved glucose uptake symmetry, likely due to enhanced motor cortex excitability and more efficient muscle activation strategies in the lower limbs. Notably, a-tDCS influenced glucose metabolism in both the more and less affected limbs. The relatively large electrode size used in this study may explain the bilateral effects, as the stimulation likely extended beyond the targeted M1 region to adjacent cortical areas.

According to this study, the observed effects of a-tDCS on lower limb strength were neither placebo-driven nor subjective—they were objectively measurable. The stimulation parameters used in this research included a current intensity greater than 2 mA, a duration of 20 minutes, and anodal stimulation over the M1. These specifications are critical for effectively modulating corticospinal excitability.

Moreover, the study highlights the importance of electrode size and placement. The relatively large electrodes used may have inadvertently stimulated contralateral cortical regions, suggesting that electrode dimensions and spatial targeting should be carefully considered when applying a-tDCS. This is particularly relevant when aiming for localized neuromodulation in motor rehabilitation protocols.

**2. Studies Investigating Quadriceps Strength in Orthopedic Conditions Before and After tDCS**

Rodrigues et al. ([78](#_ENREF_78)) conducted a study in 2022 to evaluate the effects of a-tDCS over the motor cortex combined with open kinetic chain resistance training on muscle strength and pain perception in women with patellofemoral pain (PFP). Twenty-eight women aged 18 to 30 years with PFP were randomly assigned to two groups: a-tDCS plus resistance training, and s-tDCS plus resistance training. The intervention lasted for twelve sessions, spaced 48 to 72 hours apart.

The exercise protocol consisted of three sets of 12 repetitions of knee extension at 60% of 10-repetition maximum (10RM), with one-minute rest intervals between sets. In the a-tDCS group, a 2-mA current was applied over the motor cortex for 20 minutes prior to each resistance training session. In the sham group, stimulation was discontinued after 30 seconds. Load capacity was assessed using the 10RM test before the intervention, at session 4, session 8, and after the final session. Pain perception was evaluated using the Clarke’s sign maneuver and a VAS.

Results showed that the a-tDCS plus resistance training group demonstrated significantly greater 10RM load at session 8 (p < 0.05) and post-intervention compared to the sham group. Additionally, pain perception decreased only in the a-tDCS group following the intervention. The authors concluded that this combined intervention effectively improved quadriceps strength in women with PFP. Moreover, the reduction in pain may have contributed to the observed increase in muscle strength.

This study, using stimulation parameters similar to previous research (2 mA, 20 minutes, a-tDCS over M1), demonstrated beneficial effects on quadriceps strength in a musculoskeletal population. The concurrent reduction in pain suggests that neuromodulation via tDCS may enhance muscle performance not only through cortical excitability but also by alleviating pain-related inhibition.

**3. Studies Investigating Quadriceps Strength in Healthy Athletes Before and After tDCS**

Vargas et al. ([13](#_ENREF_13)) conducted a study in 2017 to examine the modulation of isometric quadriceps strength in pre-professional female football players following a-tDCS. The aim was to assess the effect of tDCS on maximal voluntary isometric contraction (MVIC) of knee extensors in athletes actively competing at regional and national levels.

Twenty female football players aged 15–17 years, with an average of 5.2 ± 2.6 years of training experience, were randomly assigned to receive either a-tDCS or s-tDCS in a single session. The active electrode was placed over the M1 region contralateral to the dominant limb to enhance activation of the targeted quadriceps. MVIC of knee extensors in both lower limbs were measured using handheld dynamometry across five contraction sets at four time points: pre-stimulation, during a-tDCS, 30 minutes post-stimulation, and 60 minutes post-stimulation.

After a 7-day washout period, participants were reassessed with crossover stimulation. Results showed that a-tDCS led to a 2.5% increase in MVIC during stimulation, a 3.6% increase at 30 minutes, and a 4.9% increase at 60 minutes post-stimulation in the dominant limb compared to baseline. No significant changes were observed in the non-dominant limb or under sham stimulation.

The authors concluded that tDCS may serve as an effective adjunct in neuromuscular activation strategies, enhancing physical capacity and athletic performance. Furthermore, it may accelerate recovery following injury or surgery ([13](#_ENREF_13)). Given that even modest improvements in physical attributes such as strength and speed can translate into enhanced sports performance, tDCS may be a valuable tool for optimizing training outcomes ([13](#_ENREF_13), [79](#_ENREF_79)).

Maudrich et al. ([80](#_ENREF_80)) conducted a systematic review in 2022 to examine the effects of a single session of a-tDCS on specific motor performance in athletes. Optimal athletic performance results from the integration of physical and mental capacities, both of which can be trained and enhanced through targeted interventions ([81](#_ENREF_81)). The core mechanism of such interventions often involves the modulation of neural information processing ([81](#_ENREF_81)).

Evidence suggests that tDCS is a promising and feasible method for improving motor performance due to its low risk profile ([11](#_ENREF_11), [13](#_ENREF_13)) and its non-interference with task execution in both healthy and clinical populations ([13](#_ENREF_13), [39](#_ENREF_39)). Previous research has shown that tDCS can increase pain perception thresholds in healthy individuals. However, the potential of tDCS to enhance sport-specific motor performance—such as endurance, strength, and visuomotor skills (e.g., in basketball players)—remains inconclusive.

In this review, 19 studies involving a total of 258 eligible athletes were analyzed ([80](#_ENREF_80)). The findings indicated that a single session of a-tDCS applied over motor-related cortical areas may lead to improvements in sport-specific performance tasks 80. Another aspect of performance potentially influenced by M1 stimulation is pain tolerance ([78](#_ENREF_78)). Prior studies have demonstrated that M1 stimulation can elevate pain thresholds in healthy individuals ([82](#_ENREF_82)), and it has been proposed that athletes with higher pain tolerance tend to perform better ([83](#_ENREF_83)). Therefore, M1 stimulation may enhance athletic performance by reducing exercise-induced pain ([80](#_ENREF_80)).

Although no definitive conclusions can be drawn regarding the optimal stimulation protocols based on performance domain or cortical target, the findings suggest intriguing possibilities for enhancing athletic performance through anodal M1 stimulation.

While most of the reviewed studies focused on clinical populations, two investigations—by Vargas and Maudrich—specifically examined healthy athletes. These studies demonstrated that applying tDCS in combination with physical training can enhance quadriceps strength and, consequently, improve athletic performance in healthy individuals. Additionally, tDCS was shown to increase pain perception thresholds, which may contribute to improved performance by reducing exercise-induced discomfort.

**4. Studies Investigating Quadriceps Strength in Athletes with ACL Injury and ACLR Before and After tDCS**

Tohidirad et al. ([39](#_ENREF_39)) conducted a study in 2023 comparing the effects of a-tDCS with routine physiotherapy on muscular performance in athletes with anterior cruciate ligament (ACL) injury. Quadriceps dysfunction is one of the most common impairments following ACL injury in athletes ([84](#_ENREF_84)). According to the authors, due to functional changes in the motor cortex after ACL injury, conventional physiotherapy may be insufficient, and neuromodulatory interventions such as tDCS may be necessary.

Their study focused on the effects of a-tDCS applied over the M1, based on the international 10–20 EEG system, with the return electrode (cathode) placed over the contralateral supraorbital region. Thirty-four athletes with ACL injury were randomly assigned to two groups: an intervention group (a-tDCS over M1 combined with routine physiotherapy) and a control group (s-tDCS over M1 combined with routine physiotherapy).

The physiotherapy protocol included functional electrical stimulation (FES) for quadriceps retraining and strength enhancement, TENS for pain reduction, therapeutic ultrasound, and progressive exercise training. All participants received 2 mA of a-tDCS for 20 minutes during each physiotherapy session, across 10 sessions over two weeks (five sessions per week). In the sham group, stimulation was discontinued after 30 seconds.

Isokinetic knee extensor strength was measured before the intervention, immediately after, and one-month post-intervention. Results indicated a significant increase in knee extensor strength in both groups immediately and at follow-up, with the intervention group showing greater improvements than the control group ([39](#_ENREF_39)).

Unlike previous studies reviewed, this investigation applied a ten-session protocol of tDCS, using parameters consistent with earlier research—namely, 2 mA anodal stimulation over the M1 for 20 minutes. The study yielded promising results. Importantly, because it specifically targeted athletes with ACL injury, its findings are more directly aligned with the objectives of the present research.

Rush et al. ([51](#_ENREF_51)) conducted a study in 2020 to investigate the immediate effects of a-tDCS on quadriceps muscle performance in individuals with a history of ACLR. Altered quadriceps activation is a common and persistent issue following ACLR, often lasting for years post-surgery ([8](#_ENREF_8)), and is partly attributed to chronic changes in the central nervous system ([85](#_ENREF_85)). The authors hypothesized that tDCS would acutely enhance motor neuron activity by stimulating the M1.

The study employed a crossover design with 10 participants (5 male, 5 female) who were 11 months to 12 years post-ACLR. Anodal tDCS was applied over the motor cortex at a peak intensity of 2.2 mA. In both active and sham conditions, participants performed a 20-minute treadmill walking task at 2 mph with a 1% incline. Outcome measures included dynamometry, electromyography, and self-reported questionnaires (KOOS and IKDC).

Results indicated that a single session of a-tDCS did not produce immediate changes in quadriceps muscle activity, pain perception, or functional symptoms. The authors concluded that while tDCS may have induced subtle cortical changes, these were not detectable using the selected outcome measures. They suggested that more sensitive tools, such as transcranial magnetic stimulation, might better capture acute changes in corticospinal excitability. Furthermore, given that previous studies reporting beneficial effects of a-tDCS typically employed multiple treatment sessions, the authors emphasized the need for larger sample sizes and extended intervention protocols in future research.

Previous literature suggests that motor cortex excitability undergoes significant changes following ACLR. Due to this cortical reorganization, the communication between the central nervous system and the musculature surrounding the reconstructed joint becomes disrupted. As neuroplasticity increases, other brain regions outside the M1—such as the anterior cingulate gyrus and the inferior frontal gyrus—become more active to compensate for specific functional tasks. These neural pathway alterations may persist for up to six years post-ACLR ([31](#_ENREF_31)).

Participants in the study by Rush et al. ranged from 11 months to 12 years post-ACLR. Given the ongoing remodeling of neural circuits ([5](#_ENREF_5)), it appears that a single session of tDCS may be insufficient to reverse the effects of such long-term cortical changes. Future research should investigate whether applying tDCS during the acute phase of rehabilitation can help preserve or restore corticospinal excitability and prevent the documented deficits observed in ACLR patients.

Among the reviewed studies, the most closely aligned with the present investigation is the recent work by Rush et al., as it directly examined the effects of tDCS in individuals following ACLR. The stimulation parameters—2.2 mA a-tDCS over the M1 for 20 minutes—were consistent with those proposed in the current study. Although the researchers anticipated an increase in quadriceps strength, this outcome was not observed, likely due to the methodological and neurophysiological factors discussed earlier.

In summary, evidence suggests that one or more sessions of tDCS targeting the motor cortex can positively influence quadriceps muscle activity in both neurological and musculoskeletal conditions. Notably, only one study ([51](#_ENREF_51)) failed to report significant effects following a single session, which was attributed to the small sample size. Based on the reviewed literature, a protocol involving ten sessions of a-tDCS over the M1, with a current intensity of 2 mA, a duration of 20 minutes, and electrode size of 24 cm² appears to be a safe and potentially effective approach for enhancing quadriceps strength. The selected electrode size also minimizes the risk of skin irritation or burns, which may occur with smaller electrodes.

**5. Studies Investigating tDCS Stimulation Parameters Influencing Corticospinal Excitability**

Bastani et al. conducted a study in 2012 ([86](#_ENREF_86)) to examine the stimulation parameters that influence corticospinal excitability induced by tDCS. By that time, two key factors—current density and electrode size—had been identified as critical modulators of tDCS effects. Despite promising clinical outcomes using existing tDCS protocols, optimal stimulation parameters had not yet been fully standardized.

In this study, the effects of a-tDCS using three different electrode sizes (12 cm², 24 cm², and 35 cm²) were compared in 12 healthy right-handed individuals (mean age: 34.5 ± 10.32 years). A fixed current density of 0.029 mA/cm² was applied across sessions, with a constant reference electrode size of 35 cm². Each session involved 10 minutes of continuous a-tDCS, with at least 48 hours between sessions. Corticospinal excitability of the extensor carpi radialis muscle was measured before stimulation, immediately after, and at 10-, 20-, and 30-minutes post-intervention.

Findings revealed that smaller electrodes produced more focal current density and resulted in more effective and localized neuromodulation compared to larger electrodes. Follow-up comparisons showed that the 12 cm² electrode yielded the greatest increase in corticospinal excitability, followed by the 24 cm² electrode. In contrast, the 35 cm² electrode did not produce significant changes in motor evoked potentials at 20- or 30-minutes post-stimulation.

The authors concluded that reducing the size of the active electrode to one-third of the conventional size enhances spatial specificity and improves the efficacy of a-tDCS in inducing corticospinal excitability. One possible explanation is that larger electrodes may inadvertently activate adjacent cortical regions, potentially exerting inhibitory effects on the M1 ([86](#_ENREF_86)).

In a narrative review conducted by Savoury et al. ([87](#_ENREF_87)) in 2022, the authors examined the characteristics and variability of tDCS protocols across existing studies. The aim of this review was to identify which protocol modifications appear most effective in enhancing muscular strength. The authors noted that although the number of studies investigating the effects of tDCS on athletic performance has increased in recent years, considerable discrepancies remain in the literature. For example, while many studies reported that 10 to 20 minutes of tDCS led to performance improvements, others found no significant changes.

Across the reviewed studies, substantial variance was observed in the implementation of tDCS protocols. These differences included electrode placement, stimulation intensity, duration of application, and participant characteristics such as sex. Such variability may account for the conflicting results reported in the literature. The authors emphasized that numerous variables are involved in the prescription of tDCS, making direct comparisons between studies difficult. Each parameter may independently influence cortical excitability and, consequently, muscular performance.

Despite these inconsistencies, the review concluded that a-tDCS may moderately enhance maximal muscle force production and fatigue resistance. Based on the synthesis of available evidence, the authors proposed that for increasing muscular strength, a current intensity of 2 mA applied for 20 minutes is appropriate, whereas for improving muscular endurance, 1.5 mA for 10 minutes may be more effective. The reference electrode is typically positioned over the supraorbital region.

Previous studies in this section initially focused on the application of tDCS in neurological patients, reporting increased quadriceps strength following stimulation. Subsequent research extended the use of tDCS to healthy athletes and those with ACL injuries, demonstrating similar improvements in quadriceps strength. However, only one study applied tDCS in individuals following ACLR, and unlike the other studies, it did not report any increase in muscle strength. This outcome was attributed to the small sample size.

Considering the findings from earlier research on tDCS and its potential to enhance neuronal activity at the cortical level, it appears that multiple sessions of stimulation may be necessary to achieve meaningful therapeutic effects. Repeated application of a-tDCS could offer a more robust modulation of corticospinal excitability and thereby contribute to improved muscular performance in ACLR populations.

Based on the findings of Savoury, Jaberzadeh, and other prior studies on tDCS stimulation parameters, it appears that applying a-tDCS over the M1, with the cathodal electrode positioned over the supraorbital region and an electrode size of 24 cm², using a minimum current intensity of 2 mA for 20 minutes, may be appropriate for enhancing quadriceps strength when administered over ten sessions.

The hypothesis of the present study is that applying this stimulation protocol during the acute phase—when corticospinal excitability begins to decline—may help prevent the reduction in quadriceps strength commonly observed in athletes following ACLR. Quadriceps weakness often persists for months or even years after surgery, yet athletes are typically expected to return to sport within one year. This premature return may increase the risk of reinjury and lead to substantial physical, psychological, and financial consequences. If the proposed intervention proves effective, it may offer a preventive strategy to mitigate these risks and improve long-term outcomes in ACLR athletes.

**7. Project Objectives**

**7.1. General Objective**

The augmentative effect of tDCS of the motor cortex combined with routine physiotherapy on quadriceps muscle strength in athletes following ACLR

7.2. Specific Descriptive Objectives

1. To determine KOOS questionnaire scores in the intervention and control groups before and after tDCS.

2. To determine IKDC questionnaire scores in the intervention and control groups before and after tDCS.

3. To determine ACL-RSI questionnaire scores in the intervention and control groups before and after tDCS.

4. To determine pain levels using the VAS in the intervention and control groups before and after tDCS.

7.3. Specific Analytical Objectives

1. To compare the effect of motor cortex a-tDCS with sham tDCS on quadriceps strength in individuals post-ACLR, relative to the control group, before and after stimulation.

2. To compare quadriceps muscle strength in ACLR individuals before and after motor cortex stimulation with tDCS.

3. To compare central activation ratio to quantify quadriceps activation failure before and after motor cortex stimulation with tDCS.

4. To assess the correlation between a-tDCS parameters and total KOOS scores and domain scores in the intervention and control groups before and after tDCS.

5. To assess the correlation between a-tDCS and IKDC questionnaire scores in the intervention and control groups before and after tDCS.

6. To assess the correlation between a-tDCS and ACL-RSI questionnaire scores in the intervention and control groups before and after tDCS.

7. To assess the correlation between a-tDCS and VAS-reported pain scores in the intervention and control groups before and after tDCS.

8. To compare KOOS questionnaire scores in the intervention and control groups before and after tDCS.

9. To compare IKDC questionnaire scores in the intervention and control groups before and after tDCS.

10. To compare ACL-RSI questionnaire scores in the intervention and control groups before and after tDCS.

11. To compare VAS pain scores in the intervention and control groups before and after tDCS.

**7.2. Applied Objectives**

The results of this study may contribute to the refinement of tDCS protocols in rehabilitation settings aimed at improving quadriceps muscle strength. Furthermore, the findings may provide a foundation for future research into this therapeutic modality.

**8. Research Hypotheses and Questions**

1. The effect of motor cortex tDCS on quadriceps strength in athletes following ACLR differs significantly from that of the control group.

2. The effect of motor cortex tDCS on central activation ratio, used to quantify quadriceps activation failure, differs significantly between ACLR athletes and the control group.

3. KOOS questionnaire scores differ significantly between ACLR athletes and the control group.

4. IKDC questionnaire scores differ significantly between ACLR athletes and the control group.

5. ACL-RSI questionnaire scores differ significantly between ACLR athletes and the control group.

6. What is the correlation between a-tDCS and KOOS questionnaire scores?

7. What is the correlation between a-tDCS and IKDC questionnaire scores?

8. What is the correlation between a-tDCS and ACL-RSI questionnaire scores?

9. What is the correlation between a-tDCS and VAS-reported pain scores?

**9. Definition of Terms and Concepts**

**10-20 System**

**Scientific Definition**: In electroencephalography (EEG), a cap containing multiple electrodes is placed on the human scalp to record electrical activity. The 10–20 system is a standardized method for electrode placement, consisting of 19 electrodes. Each electrode is labeled according to its anatomical location—such as temporal, parietal, frontal, or occipital regions—and its corresponding hemisphere. Even-numbered electrodes (e.g., 2, 4, 6, 8) are assigned to the right hemisphere, while odd-numbered electrodes (e.g., 1, 3, 5, 7) correspond to the left hemisphere.

The term "10–20" refers to the percentage-based spacing between electrodes relative to the total circumference of the head. Electrodes near the center of the scalp are spaced 20% apart, while those closer to the periphery are spaced 10% apart. This system is illustrated in Figure 1. Additional configurations such as the 10–10 and 20–20 systems also exist, offering finer spatial resolution by reducing the inter-electrode distance—dividing the original 20% spacing into smaller 10% intervals ([88](#_ENREF_88)).

In the present study, the 10–20 system will be used for electrode placement.


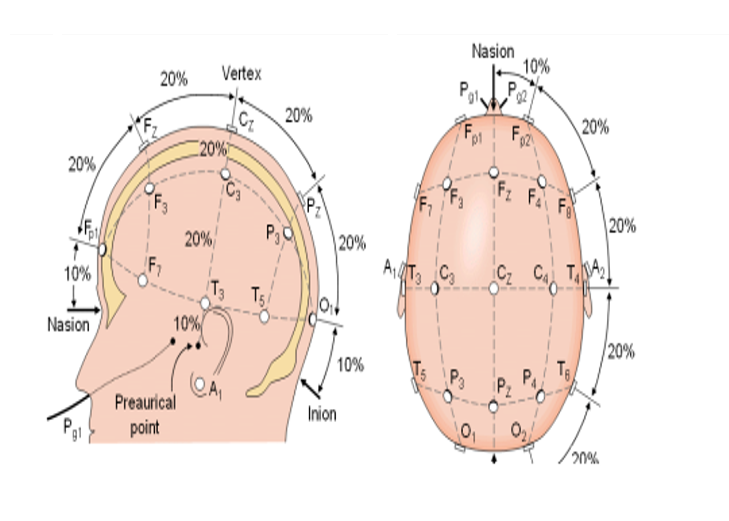


**Figure 1. The 10–20 system**

**Practical Definition:** To determine electrode placement, the 10–20 EEG system will be used. One of the key landmarks in this system is the vertex (Figure 2), located at the intersection of the line connecting the inion and nasion with the line connecting the two preauricular points. The inion is the most prominent point on the occipital bone, while the nasion is located between the forehead and the nose, at the junction of the nasal bones (Figure 3).

To identify the location of the M1, a distance equivalent to 20% of the total interaural distance is measured laterally from the vertex. This point corresponds to the C3/C4 positions in the EEG system (Figure 4) ([42](#_ENREF_42)).


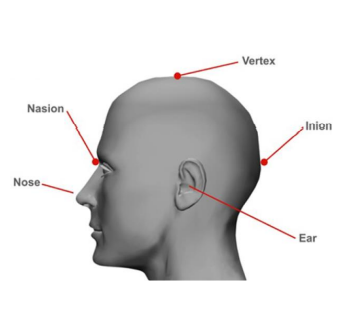

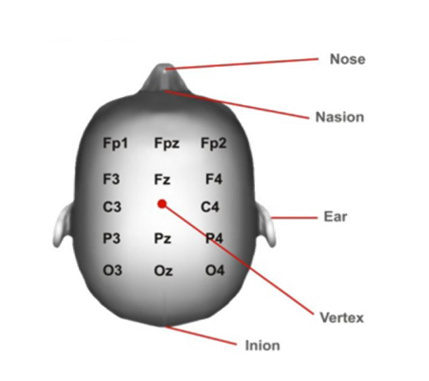


**Figure 3. Location of the inion and nasion points Figure 2. Location of the Vertex**


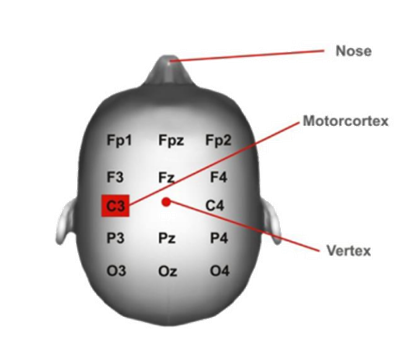


**Figure 4. Location of the primary motor cortex (M1)**

**tDCS**

**Scientific Definition:** tDCS is a neuromodulatory technique that involves the application of a low-intensity electrical current through two electrodes—an anode and a cathode—placed directly on the scalp. This method is designed to alter neuronal excitability and cortical activity ([41](#_ENREF_41)).

**Practical Definition:** In the present study, tDCS will be applied to the M1 using anodal stimulation at an intensity of 2 mA for 20 minutes per session. The intervention will be administered over ten sessions, four times per week, with the aim of enhancing corticospinal excitability in this region.

**Pain**

**Scientific Definition :**According to the International Association for the Study of Pain, pain is defined as an unpleasant sensory and emotional experience associated with actual or potential tissue damage, or described in terms of such damage ([89](#_ENREF_89)).

**Practical Definition:** In the present study, pain will be assessed using the VAS as well as the IKDC and KOOS questionnaires.

**ACLR**

**Scientific Definition:** ACLR is a surgical procedure involving the grafting of the ACL in the knee to restore its function following injury. Common graft types include patellar tendon autografts, quadriceps tendon autografts, hamstring tendon autografts, and allografts (donor tissue from cadavers).

**Practical Definition:** In the present study, ACLR is defined as reconstruction performed using quadriceps and hamstring tendon autografts, all conducted by orthopedic specialists. Eligible participants must be 2 months post-surgery at the time of enrollment.

**Arthrogenic Muscle Inhibition**

**Scientific Definition:** Arthrogenic muscle inhibition is a reflexive inhibitory mechanism that prevents full activation of a muscle, leading to reduced dynamic joint stability and subsequent muscle atrophy ([90](#_ENREF_90)).

**Practical Definition:** In the present study, the operational definition of reflexive inhibition aligns with its scientific description and will be assessed using the superimposed burst technique (SIBT). In this method, a cutaneous electrical stimulus is applied during a maximal voluntary contraction, activating previously inactive motor units. The level of voluntary muscle activation is then quantified based on the difference between the voluntary and superimposed force outputs ([91](#_ENREF_91)).

**Central Activation Ratio (CAR)**

**Scientific Definition:** Although muscle strength is the most commonly used metric for evaluating muscular performance ([59](#_ENREF_59)), it has limitations in reflecting the optimal neuromuscular capacity of a muscle ([91](#_ENREF_91)). Therefore, the CAR is employed as a more precise indicator. CAR is defined as the ratio of maximal voluntary torque to the torque generated by superimposed electrical stimulation, and it is a widely accepted measure for quantifying neuromuscular performance of the quadriceps muscle using the Superimposed Burst Technique (SIBT). Emerging evidence suggests that this technique can reveal deficits in central activation, potentially offering unique insights into the completeness of muscle function ([92](#_ENREF_92)).

**Practical Definition:** In the present study, CAR testing will be performed using FES. Participants will execute five repetitions of maximal voluntary isometric contraction (MVIC) of the quadriceps muscle, each lasting six seconds. Once a stable torque output is observed and recorded, an electrical stimulus will be applied during the MVIC to activate motor units that were not voluntarily recruited ([93](#_ENREF_93)). The stimulation protocol will consist of a burst of ten square-wave pulses at a frequency of 100 pulses per second (pps), with a pulse duration of 0.6 milliseconds. The stimulation intensity will be set at twice the motor threshold ([51](#_ENREF_51)). Five trials will be conducted with 60-second rest intervals, and the average torque values will be used to calculate voluntary activation using Equation 1.

CAR = (MVIC torque / [MVIC torque + SIB torque]) × 100 (1)

**Maximal Voluntary Isometric Contraction (MVIC)**

**Scientific Definition:** MVIC refers to the maximum voluntary force generated by a muscle, typically quantified through torque measurements ([51](#_ENREF_51)).

**Practical Definition:** In the present study, MVIC will be assessed as the average of five isometric contraction trials of the quadriceps muscle, measured using a dynamometer.

**10. Study Methodology**

**10.1. Study Design**

This study is a randomized, double-blind clinical trial in which both the participants and the assessors are blinded to group allocation.

**10.2. Target Population and Sampling Method**

The study population consists of male athletes aged 18 to 40 years ([94](#_ENREF_94)) with a history of unilateral ACLR.

**10.3. Inclusion Criteria**

1. Athletes who are at 2 months post-ACLR surgery at the time of enrollment ([8](#_ENREF_8)).

**10.4. Exclusion Criteria**

1. History of knee surgery prior to ACLR.

2. Lower limb injury within the past 6 months, excluding the current ACLR.

3. Cardiovascular disease, history of stroke, or any condition that significantly affects motor function or physical activity capacity ([51](#_ENREF_51)).

4. History of brain surgery, brain cancer, or cranial neurological disorders ([51](#_ENREF_51)).

5. Migraine ([51](#_ENREF_51)).

6. Medical history or self-reported episodes of epilepsy or seizure disorders ([13](#_ENREF_13)).

7. Presence of intracranial metal implants ([51](#_ENREF_51)).

8. Cochlear implants or other implanted electronic devices ([51](#_ENREF_51)).

9. Balance disorders or dizziness ([51](#_ENREF_51)).

10. Active skin infections or hypersensitivity to adhesive tape ([51](#_ENREF_51)).

11. Use of sedatives or medications that alter neural activity within 48 hours prior to assessment ([13](#_ENREF_13), [95](#_ENREF_95)).

**10.5. Withdrawal Criteria**

Participants will be withdrawn from the study if they experience an increase in pain during testing procedures.

**10.6. Sample Size Determination**

Due to the absence of prior studies using a similar methodology, the present study will be conducted as a pilot involving 10 participants. Based on the outcomes obtained from this initial sample, the final sample size will be calculated. Ultimately, the number of participants will be determined according to the available sample pool and the time constraints of the study.

**10.7. Study Procedures**

In this study, adult male athletes with a history of unilateral ACLR using hamstring autografts will be recruited through public announcements posted in physiotherapy clinics, outpatient centers, hospitals across Isfahan, and digital outreach via social media platforms. Participants will be selected using a non-random, convenience sampling method, based on predefined inclusion and exclusion criteria. Notably, only athletes who underwent ACLR using hamstring autografts and whose surgeries were performed using a standardized surgical technique will be eligible.

Eligible individuals will be enrolled in the study after completing an informed consent form approved by the Ethics Committee of Shahid Beheshti University of Medical Sciences. Participants will then be randomly assigned to either the intervention group or the sham stimulation group.

Prior to the start of the intervention, all participants will complete a demographic questionnaire, as well as the IKDC, ACL-RSI, and KOOS questionnaires. Pain levels will also be assessed using the VAS.

**ACL-RSI Questionnaire**

The ACL-RSI questionnaire is a psychological assessment tool designed to evaluate the mental readiness of athletes returning to sport following ACL injury ([69](#_ENREF_69)). Developed by Webster et al., this scale measures how psychological factors—including emotional responses, confidence in performance, and risk appraisal—affect an athlete’s return-to-sport decision-making. It is widely used as a predictive indicator of psychological readiness in athletes recovering from ACL injury ([96](#_ENREF_96)).

The ACL-RSI is a 12-item questionnaire, with each item rated on an 11-point scale. The instrument comprises three subdomains: (1) emotions (5 items), (2) confidence in performance (5 items), and (3) risk appraisal (2 items). The total score is calculated as a percentage of the maximum possible score across all 12 items. Higher percentage scores indicate greater psychological readiness to return to sport ([96](#_ENREF_96)).

In the present study, the Persian version of the ACL-RSI questionnaire will be used ([69](#_ENREF_69)) (Appendix 5).

**International Knee Documentation Committee (IKDC) Questionnaire**

The IKDC questionnaire is a patient-reported outcome measure specifically designed to assess knee-related clinical symptoms. It includes items related to pain intensity, daily functional activities, and overall knee performance. The instrument consists of 18 items divided into three subscales: (1) clinical symptoms (items 1–7), (2) sports activity function (items 8–17), and (3) overall knee function (item 18).

The questionnaire evaluates clinical symptoms such as pain, stiffness, swelling, joint locking, and instability, as well as functional abilities including running, jumping and landing, sudden stopping, stair navigation, standing, kneeling, sitting with a flexed knee, and rising from a chair. Scores range from 0 to 100, with higher scores indicating better functional status and milder clinical symptoms. A score of 100 reflects full participation in sports without any clinical limitations, while a score of 0 indicates severe impairment and maximal symptom burden ([97](#_ENREF_97)).

The reliability and validity of the Persian version of the IKDC questionnaire have been confirmed in ACLR patients. In the present study, the validated Persian version developed by Rahimi will be used ([98](#_ENREF_98)).

**Knee Injury and Osteoarthritis Outcome Score (KOOS)**

The KOOS questionnaire is composed of multiple subdomains, with pain and functional ability being two of its core components. In the Persian-adapted version of KOOS, pain is assessed through nine specific items. Each item is scored on a 5-point Likert scale ranging from 0 to 4, where 0 = none, 1 = mild, 2 = moderate, 3 = severe, and 4 = extreme.

Pain scoring is calculated using the average of the scores from the nine pain-related items (P1–P9), according to the standardized formula. Higher scores indicate greater levels of perceived pain ([99](#_ENREF_99)).


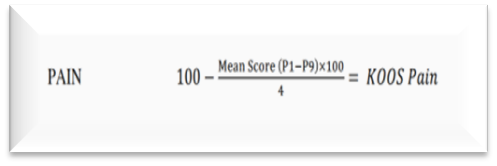


**Functional Assessment in the KOOS Questionnaire**

In the Persian-adapted version of the KOOS (Knee Injury and Osteoarthritis Outcome Score), physical function is evaluated using 17 items that cover a wide range of daily activities, such as stair climbing, rising from a seated position, and other routine movements. In addition to the daily living subscale, the questionnaire includes a sport and recreation subscale comprising five items, and a quality-of-life subscale consisting of four items.

Each item is scored on a 5-point Likert scale ranging from 0 to 4, where 0 = none, 1 = mild, 2 = moderate, 3 = severe, and 4 = extreme. The functional score is calculated using a standardized formula based on the average score across relevant items. Higher scores indicate poorer functional performance ([99](#_ENREF_99)).

ADL= Activity Daily Living, SPORT/REC= Sport/Recreation, QOL= Quality of Life

In the present study, the Persian-adapted version of the KOOS questionnaire will be used ([99](#_ENREF_99)).

**Visual Analog Scale (VAS)**

The Visual Analog Scale (VAS) is one of the most widely used tools for assessing pain intensity in clinical research. It consists of a horizontal line measuring 100 millimeters in length, where the left end (0 mm) represents "no pain" and the right end (100 mm) indicates "maximum pain intensity." Prior to treatment, participants are asked to mark the point on the line that best reflects the highest level of pain they have experienced ([100](#_ENREF_100)).

**Study Design, Randomization, and Blinding**

This study will follow a parallel-group design. After completion of the pilot phase and determination of the final sample size, eligible participants will be randomly assigned to one of two groups: (1) routine physiotherapy plus a-tDCS, or (2) routine physiotherapy plus sham tDCS (s-tDCS).

Randomization will be performed by an independent individual not involved in the study, using sealed, opaque envelopes containing each participant’s identification number and assigned group (active/sham). All assessments will be conducted by the principal investigator, while tDCS (active or sham) will be administered by a separate individual who is blinded to the study details.

To ensure double-blinding, participants will remain unaware of the nature of the intervention they receive, and the assessor will be blinded to group allocation. To achieve this, tDCS stimulation and baseline physiotherapy treatment, as well as pre- and post-intervention assessments, will be conducted independently by two separate individuals.

## Procedure

## The intervention protocol will be implemented according to the flowchart outlined below for both groups: routine physiotherapy combined with a-tDCS and routine physiotherapy combined with sham tDCS (s-tDCS).

**Quadriceps Strength Assessment Using a Dynamometer**

Quadriceps muscle strength will be measured using a dynamometer (Figure 5), a device widely utilized in previous studies ([36](#_ENREF_36), [39](#_ENREF_39), [51](#_ENREF_51)). The dynamometer enables the research team to evaluate maximal voluntary isometric contractions (MVIC) of the quadriceps muscle before and after tDCS intervention in athletes with ACLR.

Initially, isometric strength will be assessed by collecting the peak voluntary force generated by the quadriceps muscle. Participants will first perform a 5-minute warm-up on an ergometric bicycle. They will then be seated on the dynamometer with the hip and knee joints positioned at approximately 90 degrees of flexion. Stabilization straps will be secured around the waist and shoulders to minimize extraneous movement during knee extension.

The tibia will be positioned just above the ankle joint—approximately 5 cm proximal to the lateral malleolus—on the anterior surface of the lower limb, and fastened to the dynamometer arm using Velcro straps. Participants will be instructed to cross their arms over their chest throughout the test to prevent upper limb involvement ([13](#_ENREF_13)).

Once properly positioned, participants will perform two submaximal voluntary isometric contractions of the knee extensors to familiarize themselves with the testing procedure. These trials will not be recorded. Subsequently, they will practice MVIC trials (without electrical stimulation) until both the investigator and participant are confident that maximal effort is being exerted.

Following familiarization, participants will perform five maximal voluntary isometric contractions (MVIC) of the quadriceps muscle in the extension direction. Each trial will be accompanied by verbal encouragement to ensure maximal effort—participants will be verbally prompted to exert their highest possible force. A minimum rest interval of 60 seconds will be provided between trials to minimize the risk of muscular fatigue during MVIC testing.

The average torque from the five MVIC attempts will be used to quantify quadriceps strength. This procedure will be conducted prior to the tDCS intervention to establish baseline strength values. The same protocol will be repeated following completion of ten tDCS sessions to assess post-intervention changes in muscle strength.


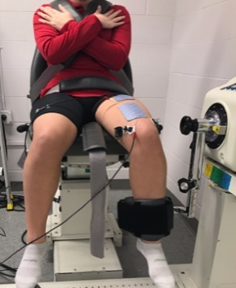
.
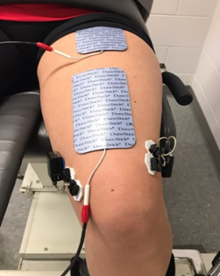


**Figure 5. Procedure for recording MVIC data and induced supra-maximal contraction in the participant**

Once each participant reaches a plateau in torque output, a supramaximal electrical stimulus will be delivered to activate motor units that were not voluntarily recruited, thereby eliciting a stronger muscle contraction. This approach is commonly used to quantify deficits in quadriceps activation following ACLR, through measurement of the Central Activation Ratio (CAR) using the Superimposed Burst Technique (SIBT) ([91](#_ENREF_91)).

For this procedure, two square-shaped electrodes (each measuring 20 cm²) will be used for electrical stimulation. One electrode will be placed proximally on the lateral aspect of the quadriceps muscle, and the other distally on the medial aspect, secured firmly with elastic bandages.

To calculate CAR, the peak voluntary torque generated immediately before the electrical stimulus is divided by the peak torque produced in response to the stimulus (i.e., the superimposed burst torque) ([51](#_ENREF_51)) (Figure 5).

**tDCS Electrode Placement Procedure**

For the tDCS intervention, electrode pads will first be moistened with normal saline solution. The anodal electrode, embedded within the pad, will then be secured using Velcro straps over the M1 region. Electrode positioning will be determined using the international 10–20 EEG system ([42](#_ENREF_42)).

The vertex (Cz) is a key anatomical landmark in this system, located at the intersection of the line connecting the inion and nasion with the line connecting the preauricular points. The inion is the most prominent point on the occipital bone, while the nasion lies between the forehead and nose, at the junction of the nasal bones.

To locate the M1, a distance equivalent to 20% of the interaural distance is measured laterally from the vertex. This corresponds to the C3 or C4 positions in the EEG system. The anodal electrode will be placed over C3 or C4, contralateral to the dominant limb, while the return (cathodal) electrode will be positioned over the supraorbital region ([101](#_ENREF_101)). Electrode placement is illustrated in Figure 6.


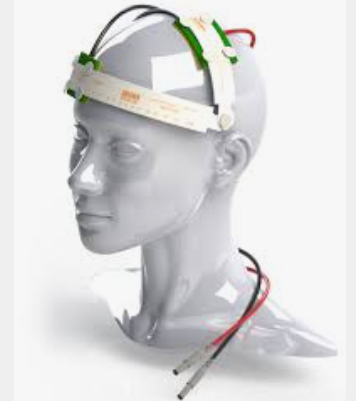


**Figure 6. Electrode placement: anode over M1 and cathode over the supraorbital area**

**tDCS Stimulation Parameters and Sham Protocol**

In this study, the anodal and cathodal electrodes will measure 24 cm² and 35 cm², respectively. The stimulation duration will be 20 minutes, with a current intensity of 2 mA. This level of current has been shown to induce changes in cortical excitability that may persist for up to 1.5 hours following stimulation ([10](#_ENREF_10)). Additionally, this intensity is considered safe and well-tolerated by participants ([42](#_ENREF_42)).

The stimulation protocol involves a gradual ramp-up of current from 0.1 mA to 2 mA over 30 seconds at the beginning of the session, followed by a gradual ramp-down over 30 seconds at the end of the 20-minute stimulation period.

For the sham intervention, the current will similarly ramp up over 30 seconds and then immediately ramp down, mimicking the sensation of active stimulation. This standardized sham procedure minimizes the likelihood of participants distinguishing between real and placebo stimulation ([50](#_ENREF_50)). Previous studies have reported that participants typically experience a tingling sensation within 10 to 20 seconds of sham stimulation, similar to that of active tDCS ([102](#_ENREF_102)).

**Intervention Protocol**

In the intervention group, participants will receive ten sessions of motor cortex tDCS, administered four times per week. The control group will follow the same schedule, but with sham stimulation. This study will be conducted under a double-blind design, ensuring that both the assessor and participants remain unaware of whether the stimulation is active or sham.

Each physiotherapy session will last 60 minutes and include the following components:

Used to retrain quadriceps muscle activation and enhance strength. Two carbon rubber electrodes (4 × 6 cm), covered with moistened sponge pads, will be placed on the proximal and distal bulk of the quadriceps muscle—one on the vastus lateralis and the other on the vastus medialis. For the hamstring muscle, two similar electrodes will be placed on its proximal and distal bulk (Figure 7).

FES parameters will include trapezoidal pulse currents with 2-second ramp-up and ramp-down times, a pulse duration of 250 microseconds, a frequency of 50 Hz, and a stimulation-to-rest ratio of 1:2 (6 seconds contraction, 12 seconds rest). Stimulation will be applied for 20 minutes per session ([39](#_ENREF_39)), with a maximum intensity of 100 mA ([94](#_ENREF_94)). The physiotherapist will adjust the intensity in each session according to the participant’s tolerance to maximize motor unit recruitment. Participants will be instructed to perform voluntary muscle contractions during the stimulation phase ([103](#_ENREF_103)).

Applied for pain reduction. Two carbon rubber electrodes (4 × 6 cm), covered with moistened sponge pads, will be placed on either side of the knee (Figure 7). TENS will be delivered in conventional mode at a frequency of 100 Hz for 20 minutes per session ([39](#_ENREF_39)).


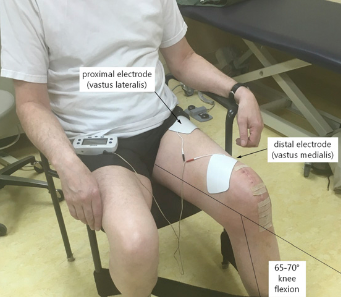


**Figure 7. Electrode placement for FES current application**

**10.8. Instruments Used in the Study**

1. Demographic Questionnaire – Used to collect background variables including age, height, weight, occupation, duration of condition, and the operated limb (Appendix 1).

2. Measuring Tape and Clinical Scale – For assessing participants’ height and weight.

3. Device used for electrical stimulation to induce SIBT

4. tDCS Device (Activedose) – Manufactured by ActivaTeK™ (Taiwan), used for transcranial direct current stimulation.

5. Dynamometer – For measuring maximal voluntary isometric contraction (MVIC) of the quadriceps muscle.

6. KOOS Questionnaire – Knee Injury and Osteoarthritis Outcome Score (Appendix 4).

7. IKDC Questionnaire – International Knee Documentation Committee form (Appendix 5).

8. ACL-RSI Questionnaire – Anterior Cruciate Ligament–Return to Sport after Injury scale (Appendix 6).

**12. Statistical Methods for Data Analysis**

Descriptive results will be presented using tables, charts, and measures of central tendency and dispersion. The normality of data distribution will be assessed using the Shapiro–Wilk test.

Given the crossover design of the study and the quantitative nature of the dependent variables, comparisons between the two interventions will include analysis of carry-over effects, sequence or period effects, and treatment effects. Depending on the distribution of the data, either the independent samples t-test or its non-parametric equivalent, the Mann–Whitney U test, will be used.

The significance level (Type I error) is set at 0.05; therefore, p-values less than 0.05 will be considered statistically significant. All statistical analyses will be performed using SPSS version 25, and graphical representations will be generated using Microsoft Excel 2018.

**13. Ethical and Human Considerations**

1. All stages of the study will respect the cultural beliefs, behaviors, and traditions of the community.

2. Participant information will remain strictly confidential.

3. Honesty and integrity will be upheld during the review and handling of all study documents.

4. A written informed consent form will be signed by all participants.

5. Participants will retain the right to withdraw from the study at any time, for any reason or without providing a reason.

6. Participation in this study will not pose any physical or psychological risk to individuals.

7. Participation will not interfere with participants’ ongoing treatment or daily life activities.

**14. Project Limitations, Potential Systematic Errors, and Mitigation Strategies**

1. Potential Equipment Malfunction:

There is a possibility of device failure during the course of the study. To mitigate this risk, all equipment will be regularly inspected and calibrated, and backup devices will be prepared in advance to ensure continuity of data collection.

2. Environmental Influences on Neural Function:

Variations in weather conditions may affect the nervous system and, consequently, the study outcomes. To minimize this confounding factor, all data collection will be conducted within a single season. Environmental conditions—including temperature, humidity, lighting, and ambient noise—will be standardized and monitored during each assessment session.

##

References

1. Majewski M, Susanne H, Klaus S. Epidemiology of athletic knee injuries: A 10-year study. The knee. 2006;13(3):184-8.

2. Gans I, Retzky JS, Jones LC, Tanaka MJ. Epidemiology of recurrent anterior cruciate ligament injuries in National Collegiate Athletic Association sports: the Injury Surveillance Program, 2004-2014. Orthopaedic journal of sports medicine. 2018;6(6):2325967118777823.

3. Salmon L, Russell V, Musgrove T, Pinczewski L, Refshauge K. Incidence and risk factors for graft rupture and contralateral rupture after anterior cruciate ligament reconstruction. Arthroscopy: The Journal of Arthroscopic & Related Surgery. 2005;21(8):948-57.

4. Luc B, Gribble PA, Pietrosimone BG. Osteoarthritis prevalence following anterior cruciate ligament reconstruction: a systematic review and numbers-needed-to-treat analysis. Journal of athletic training. 2014;49(6):806-19.

5. Lepley A, Gribble P, Thomas A, Tevald M, Sohn D, Pietrosimone B. Quadriceps neural alterations in anterior cruciate ligament reconstructed patients: a 6‐month longitudinal investigation. Scandinavian journal of medicine & science in sports. 2015;25(6):828-39.

6. Lisee C, Lepley AS, Birchmeier T, O’Hagan K, Kuenze C. Quadriceps strength and volitional activation after anterior cruciate ligament reconstruction: a systematic review and meta-analysis. Sports health. 2019;11(2):163-79.

7. Brown C, Marinko L, LaValley MP, Kumar D. Quadriceps strength after anterior cruciate ligament reconstruction compared with uninjured matched controls: a systematic review and meta-analysis. Orthopaedic Journal of Sports Medicine. 2021;9(4):2325967121991534.

8. Palmieri-Smith RM, Thomas AC, Wojtys EM. Maximizing quadriceps strength after ACL reconstruction. Clinics in sports medicine. 2008;27(3):405-24.

9. Lepley AS, Ericksen HM, Sohn DH, Pietrosimone BG. Contributions of neural excitability and voluntary activation to quadriceps muscle strength following anterior cruciate ligament reconstruction. The Knee. 2014;21(3):736-42.

10. Nitsche MA, Paulus W. Excitability changes induced in the human motor cortex by weak transcranial direct current stimulation. The Journal of physiology. 2000;527(Pt 3):633.

11. Fietsam AC, Deters JR, Workman CD, Ponto LLB, Rudroff T. Alterations in leg muscle glucose uptake and inter-limb asymmetry after a single session of tdcs in four people with multiple sclerosis. Brain sciences. 2021;11(10):1363.

12. Tanaka S, Takeda K, Otaka Y, Kita K, Osu R, Honda M, et al. Single session of transcranial direct current stimulation transiently increases knee extensor force in patients with hemiparetic stroke. Neurorehabilitation and neural repair. 2011;25(6):565-9.

13. Vargas VZ, Baptista AF, Pereira GO, Pochini AC, Ejnisman B, Santos MB, et al. Modulation of isometric quadriceps strength in soccer players with transcranial direct current stimulation: a crossover study. The Journal of Strength & Conditioning Research. 2018;32(5):1336-41.

14. Rodriguez KM, Palmieri-Smith RM, Krishnan C. How does anterior cruciate ligament reconstruction affect the functioning of the brain and spinal cord? A systematic review with meta-analysis. Journal of sport and health science. 2021;10(2):172-81.

15. Kaeding CC, Léger-St-Jean B, Magnussen RA. Epidemiology and diagnosis of anterior cruciate ligament injuries. Clinics in sports medicine. 2017;36(1):1-8.

16. Griffin LY, Albohm MJ, Arendt EA, Bahr R, Beynnon BD, DeMaio M, et al. Understanding and preventing noncontact anterior cruciate ligament injuries: a review of the Hunt Valley II meeting, January 2005. The American journal of sports medicine. 2006;34(9):1512-32.

17. Nwachukwu BU, Patel BH, Lu Y, Allen AA, Williams III RJ. Anterior cruciate ligament repair outcomes: an updated systematic review of recent literature. Arthroscopy: The Journal of Arthroscopic & Related Surgery. 2019;35(7):2233-47.

18. Carter H, Lewis G, Smith BE. Preoperative predictors for a successful return to sport following anterior cruciate ligament reconstruction (ACLR): a protocol for a systematic review and meta-analysis. BMJ open. 2021;11(12):e048295.

19. Webster KE, Feller JA. Expectations for return to preinjury sport before and after anterior cruciate ligament reconstruction. The American journal of sports medicine. 2019;47(3):578-83.

20. Dunn WR, Spindler KP, Consortium M. Predictors of activity level 2 years after anterior cruciate ligament reconstruction (ACLR) A Multicenter Orthopaedic Outcomes Network (MOON) ACLR cohort study. The American journal of sports medicine. 2010;38(10):2040-50.

21. Ardern CL, Taylor NF, Feller JA, Whitehead TS, Webster KE. Sports participation 2 years after anterior cruciate ligament reconstruction in athletes who had not returned to sport at 1 year: a prospective follow-up of physical function and psychological factors in 122 athletes. The American journal of sports medicine. 2015;43(4):848-56.

22. Lee DY, Karim SA, Chang HC. Return to sports after anterior cruciate ligament reconstruction-a review of patients with minimum 5-year follow-up. Annals Academy of Medicine Singapore. 2008;37(4):273.

23. Ardern CL, Webster KE, Taylor NF, Feller JA. Return to the preinjury level of competitive sport after anterior cruciate ligament reconstruction surgery: two-thirds of patients have not returned by 12 months after surgery. The American journal of sports medicine. 2011;39(3):538-43.

24. Adachi N, Ochi M, Uchio Y, Iwasa J, Ryoke K, Kuriwaka M. Mechanoreceptors in the anterior cruciate ligament contribute to the joint position sense. Acta Orthopaedica Scandinavica. 2002;73(3):330-4.

25. Konishi Y, Fukubayashi T, Takeshita D. Possible mechanism of quadriceps femoris weakness in patients with ruptured anterior cruciate ligament. Medicine and science in sports and exercise. 2002;34(9):1414-8.

26. Urbach D, Awiszus F. Impaired ability of voluntary quadriceps activation bilaterally interferes with function testing after knee injuries. A twitch interpolation study. International journal of sports medicine. 2002;23(04):231-6.

27. Urbach D, Nebelung W, Weiler H-T, Awiszus F. Bilateral deficit of voluntary quadriceps muscle activation after unilateral ACL tear. Medicine and science in sports and exercise. 1999;31(12):1691-6.

28. Ingersoll CD, Grindstaff TL, Pietrosimone BG, Hart JM. Neuromuscular consequences of anterior cruciate ligament injury. Clinics in sports medicine. 2008;27(3):383-404.

29. Palmieri-Smith RM, Thomas AC. A neuromuscular mechanism of posttraumatic osteoarthritis associated with ACL injury. Exercise and sport sciences reviews. 2009;37(3):147-53.

30. Pietrosimone BG, McLeod MM, Lepley AS. A theoretical framework for understanding neuromuscular response to lower extremity joint injury. Sports health. 2012;4(1):31-5.

31. Lepley AS, Grooms DR, Burland JP, Davi SM, Kinsella-Shaw JM, Lepley LK. Quadriceps muscle function following anterior cruciate ligament reconstruction: systemic differences in neural and morphological characteristics. Experimental brain research. 2019;237(5):1267-78.

32. Héroux ME, Tremblay F. Corticomotor excitability associated with unilateral knee dysfunction secondary to anterior cruciate ligament injury. Knee Surgery, Sports Traumatology, Arthroscopy. 2006;14:823-33.

33. Rosenthal MD, Moore J, Stoneman P, DeBerardino T. Neuromuscular excitability changes in the vastus medialis following anterior cruciate ligament reconstruction. Electromyography and clinical neurophysiology. 2009;49(1):43-51.

34. Rush JL, Glaviano NR, Norte GE. Assessment of quadriceps corticomotor and spinal-reflexive excitability in individuals with a history of anterior cruciate ligament reconstruction: a systematic review and meta-analysis. Sports medicine. 2021;51(5):961-90.

35. On AY, Uludağ B, Taşkiran E, Ertekin C. Differential corticomotor control of a muscle adjacent to a painful joint. Neurorehabilitation and neural repair. 2004;18(3):127-33.

36. Lepley AS, Pietrosimone B, Cormier ML. Quadriceps function, knee pain, and self-reported outcomes in patients with anterior cruciate ligament reconstruction. Journal of Athletic Training. 2018;53(4):337-46.

37. Ziemann U, Paulus W, Nitsche MA, Pascual-Leone A, Byblow WD, Berardelli A, et al. Consensus: motor cortex plasticity protocols. Brain stimulation. 2008;1(3):164-82.

38. Rahman A, Reato D, Arlotti M, Gasca F, Datta A, Parra LC, et al. Cellular effects of acute direct current stimulation: somatic and synaptic terminal effects. The Journal of physiology. 2013;591(10):2563-78.

39. Tohidirad Z, Ehsani F, Bagheri R, Jaberzadeh S. Priming effects of Anodal Transcranial Direct Current Stimulation on the effects of Conventional Physiotherapy on Balance and muscle performance in athletes with Anterior Cruciate Ligament Injury. Journal of Sport Rehabilitation. 2023;32(3):315-24.

40. Gallucci A, Lucena PH, Martens G, Thibaut A, Fregni F. Transcranial direct current stimulation to prevent and treat surgery-induced opioid dependence: a systematic review. Pain Management. 2019;9(1):93-106.

41. Zaghi S, Acar M, Hultgren B, Boggio PS, Fregni F. Noninvasive brain stimulation with low-intensity electrical currents: putative mechanisms of action for direct and alternating current stimulation. The Neuroscientist. 2010;16(3):285-307.

42. Nitsche MA, Cohen LG, Wassermann EM, Priori A, Lang N, Antal A, et al. Transcranial direct current stimulation: state of the art 2008. Brain stimulation. 2008;1(3):206-23.

43. Brunoni A, Loo C. Mood disorders. Transcranial Direct Current Stimulation in Neuropsychiatric Disorders: Springer; 2016. p. 233-44.

44. Ferrucci R, Priori A. Transcranial cerebellar direct current stimulation (tcDCS): motor control, cognition, learning and emotions. Neuroimage. 2014;85:918-23.

45. Stagg CJ, Nitsche MA. Physiological basis of transcranial direct current stimulation. The Neuroscientist. 2011;17(1):37-53.

46. Nitsche M, Fricke K, Henschke U, Schlitterlau A, Liebetanz D, Lang N, et al. Pharmacological modulation of cortical excitability shifts induced by transcranial direct current stimulation in humans. The Journal of physiology. 2003;553(1):293-301.

47. Nitsche MA, Seeber A, Frommann K, Klein CC, Rochford C, Nitsche MS, et al. Modulating parameters of excitability during and after transcranial direct current stimulation of the human motor cortex. The Journal of physiology. 2005;568(1):291-303.

48. Stagg CJ, Best JG, Stephenson MC, O'Shea J, Wylezinska M, Kincses ZT, et al. Polarity-sensitive modulation of cortical neurotransmitters by transcranial stimulation. Journal of Neuroscience. 2009;29(16):5202-6.

49. Kim S, Stephenson MC, Morris PG, Jackson SR. tDCS-induced alterations in GABA concentration within primary motor cortex predict motor learning and motor memory: a 7 T magnetic resonance spectroscopy study. Neuroimage. 2014;99:237-43.

50. Gandiga PC, Hummel FC, Cohen LG. Transcranial DC stimulation (tDCS): a tool for double-blind sham-controlled clinical studies in brain stimulation. Clinical neurophysiology. 2006;117(4):845-50.

51. Rush JL, Lepley LK, Davi S, Lepley AS. The immediate effects of transcranial direct current stimulation on quadriceps muscle function in individuals with a history of anterior cruciate ligament reconstruction: a preliminary investigation. Journal of Sport Rehabilitation. 2020;29(8):1121-30.

52. Kuenze C, Pietrosimone B, Lisee C, Rutherford M, Birchmeier T, Lepley A, et al. Demographic and surgical factors affect quadriceps strength after ACL reconstruction. Knee Surgery, Sports Traumatology, Arthroscopy. 2019;27(3):921-30.

53. Lepley LK. Deficits in quadriceps strength and patient-oriented outcomes at return to activity after ACL reconstruction: a review of the current literature. Sports health. 2015;7(3):231-8.

54. Kaur M, Ribeiro DC, Theis J-C, Webster KE, Sole G. Movement patterns of the knee during gait following ACL reconstruction: a systematic review and meta-analysis. Sports medicine. 2016;46(12):1869-95.

55. DeFroda SF, ODonnell RM, Fadale PD, Owens BD, Fleming BC. The role of magnetic resonance imaging in evaluating postoperative ACL reconstruction healing and graft mechanical properties: a new criterion for return to play? The Physician and Sportsmedicine. 2021;49(2):123-9.

56. Grindem H, Snyder-Mackler L, Moksnes H, Engebretsen L, Risberg MA. Simple decision rules can reduce reinjury risk by 84% after ACL reconstruction: the Delaware-Oslo ACL cohort study. British journal of sports medicine. 2016;50(13):804-8.

57. Kuenze CM, Foot N, Saliba SA, Hart JM. Drop-landing performance and knee-extension strength after anterior cruciate ligament reconstruction. Journal of athletic training. 2015;50(6):596-602.

58. Lepley LK, Palmieri-Smith RM. Quadriceps strength, muscle activation failure, and patient-reported function at the time of return to activity in patients following anterior cruciate ligament reconstruction: a cross-sectional study. journal of orthopaedic & sports physical therapy. 2015;45(12):1017-25.

59. Blackburn JT, Pietrosimone B, Harkey MS, Luc BA, Pamukoff DN. Quadriceps Function and Gait Kinetics after Anterior Cruciate Ligament Reconstruction. Medicine and science in sports and exercise. 2016;48(9):1664-70.

60. Xergia SA, Pappas E, Zampeli F, Georgiou S, Georgoulis AD. Asymmetries in functional hop tests, lower extremity kinematics, and isokinetic strength persist 6 to 9 months following anterior cruciate ligament reconstruction. Journal of Orthopaedic & Sports Physical Therapy. 2013;43(3):154-62.

61. He X, Huang WY, Leong HT, Qiu JH, Ma CC, Fu S-C, et al. Decreased passive muscle stiffness of vastus medialis is associated with poorer quadriceps strength and knee function after anterior cruciate ligament reconstruction. Clinical Biomechanics. 2021;82:105289.

62. Buckthorpe M, Danelon F, La Rosa G, Nanni G, Stride M, Della Villa F. Recommendations for hamstring function recovery after ACL reconstruction. Sports Medicine. 2021;51(4):607-24.

63. Setuain I, Izquierdo M, Idoate F, Bikandi E, Gorostiaga EM, Aagaard P, et al. Differential effects of 2 rehabilitation programs following anterior cruciate ligament reconstruction. Journal of sport rehabilitation. 2017;26(6):544-55.

64. Snow BJ, Wilcox JJ, Burks RT, Greis PE. Evaluation of muscle size and fatty infiltration with MRI nine to eleven years following hamstring harvest for ACL reconstruction. JBJS. 2012;94(14):1274-82.

65. van Melick N, van der Weegen W, van der Horst N. Quadriceps and hamstrings strength reference values for athletes with and without anterior cruciate ligament reconstruction who play popular pivoting sports, including soccer, basketball, and handball: a scoping review. journal of orthopaedic & sports physical therapy. 2022;52(3):142-55.

66. Maestroni L, Read P, Turner A, Korakakis V, Papadopoulos K. Strength, rate of force development, power and reactive strength in adult male athletic populations post anterior cruciate ligament reconstruction-A systematic review and meta-analysis. Physical Therapy in Sport. 2021;47:91-104.

67. Cronström A, Tengman E, Häger CK. Return to sports: a risky business? A systematic review with meta-analysis of risk factors for graft rupture following ACL reconstruction. Sports medicine. 2023;53(1):91-110.

68. Vutescu ES, Orman S, Garcia-Lopez E, Lau J, Gage A, Cruz Jr AI. Psychological and social components of recovery following anterior cruciate ligament reconstruction in young athletes: a narrative review. International Journal of Environmental Research and Public Health. 2021;18(17):9267.

69. Pirayeh N, Razavi F, Behdarvandan A, Mostafaee N. Anterior cruciate ligament-return to sport after injury scale: reliability and validity of the persian version. Journal of sport rehabilitation. 2023;32(4):369-75.

70. Belk JW, Kraeutler MJ, Marshall HA, Goodrich JA, McCarty EC. Quadriceps tendon autograft for primary anterior cruciate ligament reconstruction: a systematic review of comparative studies with minimum 2-year follow-up. Arthroscopy: The Journal of Arthroscopic & Related Surgery. 2018;34(5):1699-707.

71. Winkler PW, Vivacqua T, Thomassen S, Lovse L, Lesniak BP, Getgood AM, et al. Quadriceps tendon autograft is becoming increasingly popular in revision ACL reconstruction. Knee Surgery, Sports Traumatology, Arthroscopy. 2022;30(1):149-60.

72. Kurz A, Evaniew N, Yeung M, Samuelsson K, Peterson D, Ayeni OR. Credibility and quality of meta-analyses addressing graft choice in anterior cruciate ligament reconstruction: a systematic review. Knee Surgery, Sports Traumatology, Arthroscopy. 2017;25(2):538-51.

73. Sajovic M, Strahovnik A, Dernovsek MZ, Skaza K. Quality of life and clinical outcome comparison of semitendinosus and gracilis tendon versus patellar tendon autografts for anterior cruciate ligament reconstruction: an 11-year follow-up of a randomized controlled trial. The American journal of sports medicine. 2011;39(10):2161-9.

74. Perriman A, Leahy E, Semciw AI. The effect of open-versus closed-kinetic-chain exercises on anterior tibial laxity, strength, and function following anterior cruciate ligament reconstruction: a systematic review and meta-analysis. journal of orthopaedic & sports physical therapy. 2018;48(7):552-66.

75. Smith AH, Capin JJ, Zarzycki R, Snyder-Mackler L. Athletes with bone-patellar tendon-bone autograft for anterior cruciate ligament reconstruction were slower to meet rehabilitation milestones and return-to-sport criteria than athletes with hamstring tendon autograft or soft tissue allograft: secondary analysis from the ACL-SPORTS trial. journal of orthopaedic & sports physical therapy. 2020;50(5):259-66.

76. Herbawi F, Lozano-Lozano M, Lopez-Garzon M, Postigo-Martin P, Ortiz-Comino L, Martin-Alguacil JL, et al. A systematic review and meta-analysis of strength recovery measured by isokinetic dynamometer technology after anterior cruciate ligament reconstruction using quadriceps tendon autografts vs. hamstring tendon autografts or patellar tendon autografts. International Journal of Environmental Research and Public Health. 2022;19(11):6764.

77. Hummel FC, Voller B, Celnik P, Floel A, Giraux P, Gerloff C, et al. Effects of brain polarization on reaction times and pinch force in chronic stroke. BMC neuroscience. 2006;7(1):73.

78. Rodrigues GM, Paixão A, Arruda T, de Oliveira BRR, Neto GAM, Neto SRM, et al. Anodal transcranial direct current stimulation increases muscular strength and reduces pain perception in women with patellofemoral pain. The Journal of Strength & Conditioning Research. 2022;36(2):371-8.

79. Banissy MJ, Muggleton NG. Transcranial direct current stimulation in sports training: potential approaches. Frontiers in human neuroscience. 2013;7:129.

80. Maudrich T, Ragert P, Perrey S, Kenville R. Single-session anodal transcranial direct current stimulation to enhance sport-specific performance in athletes: A systematic review and meta-analysis. Brain Stimulation. 2022;15(6):1517-29.

81. Chang M, Büchel D, Reinecke K, Lehmann T, Baumeister J. Ecological validity in exercise neuroscience research: A systematic investigation. European Journal of Neuroscience. 2022;55(2):487-509.

82. Vaseghi B, Zoghi M, Jaberzadeh S. Does anodal transcranial direct current stimulation modulate sensory perception and pain? A meta-analysis study. Clinical Neurophysiology. 2014;125(9):1847-58.

83. Astokorki A, Mauger AR. Tolerance of exercise‐induced pain at a fixed rating of perceived exertion predicts time trial cycling performance. Scandinavian journal of medicine & science in sports. 2017;27(3):309-17.

84. Iossifidou AN, Baltzopoulos V. Inertial effects on moment development during isokinetic concentric knee extension testing. Journal of Orthopaedic & Sports Physical Therapy. 2000;30(6):317-27.

85. Needle AR, Lepley AS, Grooms DR. Central nervous system adaptation after ligamentous injury: a summary of theories, evidence, and clinical interpretation. Sports medicine. 2017;47(7):1271-88.

86. Bastani A, Jaberzadeh S. a-tDCS differential modulation of corticospinal excitability: the effects of electrode size. Brain stimulation. 2013;6(6):932-7.

87. Savoury RB, Kibele A, Power KE, Herat N, Alizadeh S, Behm DG. Reduced isometric knee extensor force following anodal transcranial direct current stimulation of the ipsilateral motor cortex. Plos one. 2023;18(1):e0280129.

88. Sun S. Extreme energy difference for feature extraction of EEG signals. Expert Systems with Applications. 2010;37(6):4350-7.

89. Treede R-D. The International Association for the Study of Pain definition of pain: as valid in 2018 as in 1979, but in need of regularly updated footnotes. Pain reports. 2018;3(2):e643.

90. Patel HH, Berlinberg EJ, Nwachukwu B, Williams III RJ, Mandelbaum B, Sonkin K, et al. Quadriceps weakness is associated with neuroplastic changes within specific corticospinal pathways and brain areas after anterior cruciate ligament reconstruction: theoretical utility of motor imagery-based brain-computer interface technology for rehabilitation. Arthroscopy, Sports Medicine, and Rehabilitation. 2023;5(1):e207-e16.

91. Gilfeather D, Norte G, Ingersoll CD, Glaviano NR. Central activation ratio is a reliable measure for gluteal neuromuscular function. Journal of Sport Rehabilitation. 2019;29(7):956-62.

92. Ward SH, Blackburn JT, Padua DA, Stanley LE, Harkey MS, Luc-Harkey BA, et al. Quadriceps neuromuscular function and jump-landing sagittal-plane knee biomechanics after anterior cruciate ligament reconstruction. Journal of athletic training. 2018;53(2):135-43.

93. Mizner RL, Stevens JE, Snyder-Mackler L. Voluntary activation and decreased force production of the quadriceps femoris muscle after total knee arthroplasty. Physical Therapy. 2003;83(4):359-65.

94. Moran U, Gottlieb U, Gam A, Springer S. Functional electrical stimulation following anterior cruciate ligament reconstruction: a randomized controlled pilot study. Journal of neuroengineering and rehabilitation. 2019;16:1-9.

95. Angius L, Pageaux B, Hopker J, Marcora SM, Mauger AR. Transcranial direct current stimulation improves isometric time to exhaustion of the knee extensors. Neuroscience. 2016;339:363-75.

96. Webster KE, Feller JA, Lambros C. Development and preliminary validation of a scale to measure the psychological impact of returning to sport following anterior cruciate ligament reconstruction surgery. Physical therapy in sport. 2008;9(1):9-15.

97. Irrgang JJ, Anderson AF, Boland AL, Harner CD, Kurosaka M, Neyret P, et al. Development and validation of the international knee documentation committee subjective knee form. The American journal of sports medicine. 2001;29(5):600-13.

98. Rahimi A, Nowrouzi A, Sohani SM. The validity and reliability of the persian version of the international knee documentation committee (IKDC) questionnaire in Iranian patients after acl and meniscal surgeries. Archives of Rehabilitation. 2013;14(2):116-24.

99. Salavati M, Mazaheri M, Negahban H, Sohani S, Ebrahimian M, Ebrahimi I, et al. Validation of a Persian-version of Knee injury and Osteoarthritis Outcome Score (KOOS) in Iranians with knee injuries. Osteoarthritis and Cartilage. 2008;16(10):1178-82.

100. Jensen MP, Chen C, Brugger AM. Interpretation of visual analog scale ratings and change scores: a reanalysis of two clinical trials of postoperative pain. The Journal of pain. 2003;4(7):407-14.

101. Madhavan S, Stinear JW. Focal and bidirectional modulation of lower limb motor cortex using anodal transcranial direct current stimulation. Brain stimulation. 2010;3(1):42-50.

102. Kaski D, Quadir S, Patel M, Yousif N, Bronstein AM. Enhanced locomotor adaptation aftereffect in the “broken escalator” phenomenon using anodal tDCS. Journal of neurophysiology. 2012;107(9):2493-505.

103. Taradaj J, Halski T, Kucharzewski M, Walewicz K, Smykla A, Ozon M, et al. The effect of neuromuscular electrical stimulation on quadriceps strength and knee function in professional soccer players: return to sport after ACL reconstruction. BioMed research international. 2013;2013(1):802534.
