# Supplementary material for: Integrating multi-session transcranial direct current stimulation with routine physical therapy to improve quadriceps strength and activation in athletes during subacute recovery following ACL reconstruction: A double-blind RCT
Source: PLoS One. 2026 Jun 11;21(6):e0345947. doi: 10.1371/journal.pone.0345947 (PMC13257960; doi:10.1371/journal.pone.0345947)
Supplement: S2 File — (DOCX) [file pone.0345947.s002.docx]

Inclusivity in global research

PLOS’ policy on inclusivity in global research aims to improve transparency in the reporting of research performed outside of researchers’ own country or community and ensures that PLOS publications reporting global research adhere to high standards for research ethics and authorship. Authors of relevant research articles may be asked to complete the questionnaire below, which outlines ethical, cultural, and scientific considerations specific to inclusivity in global research. This questionnaire may be requested when researchers have travelled to a different country to conduct research, if research uses samples collected in another country, research with Indigenous populations or their lands, or if research is on cultural artefacts. Researchers travelling to another country solely to use laboratory equipment will not normally be required to complete the questionnaire. However, the questionnaire can be requested at the journal’s discretion for any submission – if you have been requested to complete this questionnaire by the PLOS journal you submitted to, please do so.

Please complete the questionnaire below and include this as a Supporting Information file with your manuscript. Note that if your paper is accepted for publication, this checklist will be published with your article in the supporting information files. Please ensure that you reference the checklist in the main body of your manuscript. We suggest adding a subsection ‘Inclusivity in global research’ to your Methods section and adding the following sentence: “Additional information regarding the ethical, cultural, and scientific considerations specific to inclusivity in global research is included in the Supporting Information (SX Checklist)”

The questions have been designed to be applicable to a wide range of study types, and there are subsections for both human subjects research and non-human subjects research. If any of the questions are not relevant to your research please mark them as “N/A” as appropriate.

**Ethical considerations, permits and authorship**

*This section is applicable to all research types.*

Provide details as to who granted permissions and/or consent for the study to take place in the Methods section of your manuscript. This should include the names of **all** ethics boards, governmental organizations, community leaders or other bodies that provided approval for the study. If individuals provided approval refer to these people by their role or title but do not list their name(s).

Reported on page number: This study was registered in the Iranian Registry of Clinical Trials (IRCT) under registration number IRCT20231113060048N1 (Registered on December 28, 2023). Ethical approval was obtained from the Ethics Committee of Shahid Beheshti University of Medical Sciences (approval code: IR.SBMU.RETECH.REC.1402.163), and the study was conducted in accordance with the university’s ethical guidelines for interventional research. Written informed consent was obtained from all participants. This research forms part of a PhD dissertation in physical therapy by N.H.E., approved under registration number 43005662. Shahid Beheshti University of Medical Sciences was not involved in any stage of the study, including its design, implementation, analysis, or submission.

If there were any deviations from the study protocol after approval was obtained please provide details of these changes in the Methods section of your manuscript.
Did this study involve local collaborators that are residents of the country where the research was conducted or members of the community studied? If you do not have any authors from said communities, please provide an explanation for this below.

Reported on page number:

Isometric quadriceps strength was evaluated by measuring the Normalized Peak Torque (NPT) generated during MVIC, using an isokinetic dynamometer (Biodex System 3; Biodex Medical Systems, Inc., Shirley, NY). This timing —week 10 instead of the originally planned week 8—was selected to enhance measurement reliability and reduce the confounding effects of early postoperative factors such as pain, swelling, and arthrogenic muscle inhibition (manuscript page 16).

Although the original protocol specified inclusion of hamstring autograft recipients only, logistical constraints and graft availability led to the inclusion of both allograft and autograft cases. This change was necessary to ensure adequate participant recruitment due to limited access to hamstring grafts at the study site. Of these, 14 underwent ACL reconstruction using allografts, and 6 received hamstring autografts. (manuscript page 8).

Everyone listed as an author should meet PLOS’ criteria for authorship and all individuals who meet these criteria should be included in the author byline, rather than the acknowledgements. For further information please see the journal’s Authorship Policy.

**Human subjects research (e.g. health research, medical research, cross-cultural psychology)**

Did you obtain written informed consent from a representative of the local community or region before the research took place? How did you establish who speaks for the community? Details of written informed consent obtained from study participants should be reported separately in the Methods section of your manuscript.

The study was conducted in a licensed physiotherapy center under the supervision of the Deputy of Treatment at the Ministry of Health. All quadriceps strength and activation measurements were performed using a Biodex isokinetic dynamometer at the Human Movement Laboratory of the University of Isfahan. These measurements were conducted by one of the co-authors, who was responsible for the data collection and is affiliated with the university. Written informed consent was obtained from all individual participants prior to enrollment. No additional community-level consent was required, as both the clinical and laboratory settings were authorized institutions operating under national regulatory oversight.

How did members of the local community provide input on the aims of the research investigation, its methodology, and its anticipated outcome(s)?

The study was designed by the research team based on a review of clinical needs and existing literature. While members of the local community were not directly involved in the study design, the research questions and methodology were informed by prior clinical experience, patient feedback, and the needs identified by the physiotherapy center where the study was conducted.

When engaging with the local community, how did you ensure that the informed consent documents and other materials could be understood by local stakeholders?

Will the findings of the research be made available in an understandable format to stakeholders in the community where the study was conducted (e.g. via a presentation, summary report, copies of publications, etc.)? Please provide details of how this will be achieved.

The informed consent documents were written in Persian, the native language of all participants, using clear and non-technical language. The research team provided verbal explanations and answered participants’ questions to ensure full understanding before consent was obtained.

**Non-human subjects research using specimens/ animals collected as part of the study, or those housed in archival collections. Examples include archaeology, paleontology, botany and zoology.**

Did the permission you obtained from a local authority to perform the study include an agreement on access to outputs and benefit sharing? This may include procedures to enable fair distribution of the benefits and resources arising from the research performed. Please include any details of Prior Informed Consent and Benefit Sharing Agreements obtained. These may be required by field-specific regulations, for example the Convention on Biological Diversity (CBD) and the associated Nagoya Protocol.

This study did not involve non-human specimens, animals, or materials from archival collections. Therefore, prior informed consent and benefit-sharing agreements were not applicable.

If the material used in your study was imported, please A) provide the year it was imported and B) indicate whether permits were obtained to import/export the materials used, C) provide details of any permits obtained. If this information is not available, please indicate this.

No imported materials were used in this study. Therefore, no import/export permits were required.

If you used archival specimens, please state how the material used in your study was acquired by the institute it is held in and provide details of any permits obtained for the original excavations/ sample collection. If this information is not available, please indicate this.

This study did not involve the use of archival specimens. Therefore, no information on acquisition or excavation permits is applicable.

How was the potential cultural significance of the materials collected in your study to local communities considered in your research design? Were Indigenous peoples and/or local researchers and institutions involved with archaeological excavations / collection of specimens? If so, please provide a description of their involvement.

This study did not involve the collection of materials with potential cultural significance, nor did it involve archaeological excavations or the collection of specimens. Therefore, no involvement of Indigenous peoples or local institutions in this context was required.

If your manuscript includes photographs of human remains please indicate whether authors obtained permission from descendants or affiliated cultural communities to do so.

This manuscript includes a photograph of a person in the laboratory setting. Written informed consent for publication of the image, including permission to display the individual’s identifiable facial features, was obtained prior to submission.
