# Supplementary material for: Integrating multi-session transcranial direct current stimulation with routine physical therapy to improve quadriceps strength and activation in athletes during subacute recovery following ACL reconstruction: A double-blind RCT
Source: PLoS One. 2026 Jun 11;21(6):e0345947. doi: 10.1371/journal.pone.0345947 (PMC13257960; doi:10.1371/journal.pone.0345947)
Supplement: S3 File — (DOCX) [file pone.0345947.s003.docx]

|  | Section/topic | No | CONSORT 2025 checklist item description | Reported on page no. |
| --- | --- | --- | --- | --- |
|  | **Title and abstract** | | |  |
|  | Title and structured abstract | 1a | Identification as a randomised  trial | Title Page and page 1 |
|  |  | 1b | Structured summary of the trial design, methods, results, and conclusions | Page 1 and 2 |
|  | **Open science** | | |  |
|  | Trial registration | 2 | Name of trial registry, identifying number (with URL) and date of registration | Title Page |
|  | Protocol and statistical analysis plan | 3 | Where the trial protocol and statistical analysis plan can be accessed | Supplementary File 1 and Statistical Analysis Plan available in protocol and pp. 17–22 of main text. |
|  | Data sharing | 4 | Where and how the individual de-identified participant data (including data dictionary), statistical code and any other materials can be accessed | Data are in tables page 19 and 21; See Data Availability page 32 for raw data access details. |
|  | Funding and conflicts of interest | 5a | Sources of funding and other support (eg, supply of drugs), and role of funders in the design, conduct, analysis and reporting of the trial | Page 33 of the main manuscript |
|  |  | 5b | Financial and other conflicts of interest of the manuscript authors | Page 33 of the main manuscript |
|  | **Introduction** | | |  |
|  | Background and rationale | 6 | Scientific background and rationale | Page 3-6 of the manuscript. |
|  | Objectives | 7 | Specific objectives related to benefits and harms | Pages 5 and 6 |
|  | **Methods** | | |  |
|  | Patient and public involvement | 8 | Details of patient or public involvement in the design, conduct and reporting of the trial | Page 6, lines 114-115 of the manuscript. |
|  | Trial design | 9 | Description of trial design including type of trial (eg, parallel group, crossover), allocation ratio, and framework (eg, superiority, equivalence, non-inferiority, exploratory) | Page 6, lines 117-122 of the manuscript |
|  |  | 10 | Important changes to the trial after it commenced including any outcomes or analyses that were not prespecified, with reason | Title page – “revised title”, Page 5 Lines 92-98 and Page 7, lines  143-146 |
|  | Trial setting | 11 | Settings (eg, community, hospital) and locations (eg, countries, sites) where the trial was conducted | Page 6 and 7, lines 125-130 of the manuscript |
|  | Eligibility criteria | 12a | Eligibility criteria for participants | Page 7, lines 132-147 of the manuscript |
|  |  | 12b | If applicable, eligibility criteria for sites and for individuals delivering the interventions (eg, surgeons, physiotherapists) | Page 7, lines 141-142 |
|  | Intervention and comparator | 13 | Intervention and comparator with sufficient details to allow replication. If relevant, where additional materials describing the intervention and comparator (eg, intervention manual) can be accessed | pages 8-12 of the manuscript |
|  | Outcomes | 14 | Prespecified primary and secondary outcomes, including the specific measurement variable (eg, systolic blood pressure), analysis metric (eg, change from baseline, final value, time to event), method of aggregation (eg, median, proportion), and time point for each outcome | pages 12-14 of the manuscript |
|  | Harms | 15 | How harms were defined and assessed (eg, systematically, non-systematically) | Page 15 lines 305-310 of the manuscript |
|  | Sample size | 16a | How sample size was determined, including all assumptions supporting the sample size calculation | Page 16, lines 311-322 of the manuscript |
|  |  | 16b | Explanation of any interim analyses and stopping guidelines | Page 15, lines 322-323 of the manuscript |
|  | Randomisation: |  |  | Page 16 lines 327 of the manuscript |
|  | Sequence generation | 17a | Who generated the random allocation sequence and the method used |  |
|  |  | 17b | Type of randomisation and details of any restriction (eg, stratification, blocking and block size) | Page 16, line 326 of the manuscript |
|  |  |  |  |  |
|  | Allocation concealment mechanism | 18 | Mechanism used to implement the random allocation sequence (eg, central computer/telephone; sequentially numbered, opaque, sealed containers), describing any steps to conceal the sequence until interventions were assigned | Page 16, line 329-331 of the manuscript |
|  | Implementation | 19 | Whether the personnel who enrolled and those who assigned participants to the interventions had access to the random allocation sequence | Page 16, Lines 332-334 |
|  | Blinding | 20a | Who was blinded after assignment to interventions (eg, participants, care providers, outcome assessors, data analysts) | Page 16 |
|  |  | 20b | If blinded, how blinding was achieved and description of the similarity of interventions | Page 16 |
|  | Statistical methods | 21a | Statistical methods used to compare groups for primary and secondary outcomes, including harms | Page 17 |
|  |  | 21b | Definition of who is included in each analysis (eg, all randomised participants), and in which group | Page 17 |
|  |  | 21c | How missing data were handled in the analysis | Page 17 |
|  |  | 21d | Methods for any additional analyses (eg, subgroup and sensitivity analyses), distinguishing prespecified from post hoc | Page 17 |
|  | **Results** | | |  |
|  | Participant flow, including flow diagram | 22a | For each group, the numbers of participants who were randomly assigned, received intended intervention, and were analysed for the primary outcome | Page 18 and Fig 4 |
|  |  | 22b | For each group, losses and exclusions after randomisation, together with reasons | Page 18 |
|  | Recruitment | 23a | Dates defining the periods of recruitment and follow-up for outcomes of benefits and harms | Page 18, Lines 368-369 |
|  |  | 23b | If relevant, why the trial ended or was stopped | Not applicable – trial completed as planned |
|  | Intervention and comparator delivery | 24a | Intervention and comparator as they were actually administered (eg, where appropriate, who delivered the intervention/comparator, how participants adhered, whether they were delivered as intended (fidelity)) | Page 18, Lines 372-380 |
|  |  | 24b | Concomitant care received during the trial for each group | Page 18, Lines 377-380 |
|  | Baseline data | 25 | A table showing baseline demographic and clinical characteristics for each group | Page 19 |
|  | Numbers analysed,  outcomes and estimation | 26 | For each primary and secondary outcome, by group:  ● the number of participants included in the analysis  ● the number of participants with available data at the outcome time point  ● result for each group, and the estimated effect size and its precision (such as 95% confidence interval)  ● for binary outcomes, presentation of both absolute and relative effect size | Pages 19-22  Page 19-22  Page 19-22  Pages 19-22  Not applicable – no binary outcomes assessed |
|  | Harms | 27 | All harms or unintended events in each group | Page 18, line 380 of the manuscript |
|  | Ancillary analyses | 28 | Any other analyses performed, including subgroup and sensitivity analyses, distinguishing pre-specified from post hoc | Page 22 and 23, lines 441-465 of the manuscript |
|  | **Discussion** | | | Pages 23-31 |
|  | Interpretation | 29 | Interpretation consistent with results, balancing benefits and harms, and considering other relevant evidence | Pages 23-31 |
|  | Limitations | 30 | Trial limitations, addressing sources of potential bias, imprecision, generalisability, and, if relevant, multiplicity of analyses | Page 32, Lines 653-669 |

Citation: Hopewell S, Chan AW, Collins GS, Hróbjartsson A, Moher D, Schulz KF, et al. CONSORT 2025 Statement: updated guideline for reporting randomised trials. BMJ. 2025; 388:e081123. <https://dx.doi.org/10.1136/bmj-2024-081123>
© 2025 Hopewell et al. This is an Open Access article distributed under the terms of the Creative Commons Attribution License (<https://creativecommons.org/licenses/by/4.0/>), which permits unrestricted use, distribution, and reproduction in any medium, provided the original work is properly cited.

*We strongly recommend reading this statement in conjunction with the CONSORT 2025 Explanation and Elaboration and/or the CONSORT 2025 Expanded Checklist for important clarifications on all the items. We also recommend reading relevant CONSORT extensions. See [www.consort-spirit.org](http://www.consort-spirit.org).
